# Molecular phylogeny of the Tiliae group (*Inocybe* sect. *Marginatae*) and delimitation of the group with new discoveries

**DOI:** 10.3897/mycokeys.139.204873

**Published:** 2026-08-25

**Authors:** Yu-Guang Fan, Yan-Ru Xu, Ying Li, Cheng Chang, Lun-Sha Deng, Wen-Jie Yu

**Affiliations:** 1 School of Public Health, Key Laboratory of Tropical Translational Medicine of Ministry of Education, Hainan Medical University, Haikou, 571199, China Engineering Research Center of Tropical Medicine Innovation and Transformation of Ministry of Education, Hainan Provincial Key Laboratory of Research and Development on Tropical Herbs, International Center for Aging and Cancer, School of Pharmacy, Hainan Medical University Haikou China; 2 Engineering Research Center of Tropical Medicine Innovation and Transformation of Ministry of Education, Hainan Provincial Key Laboratory of Research and Development on Tropical Herbs, International Center for Aging and Cancer, School of Pharmacy, Hainan Medical University, Haikou, 571199, China School of Public Health, Key Laboratory of Tropical Translational Medicine of Ministry of Education, Hainan Medical University Haikou China; 3 College of Biological and Food Engineering, Guangxi Science and Technology Normal University, Laibin, Guangxi 546100, China College of Biological and Food Engineering, Guangxi Science and Technology Normal University Laibin China

**Keywords:** *
Agaricales
*, China, *
Inocybaceae
*, species diversity, taxonomy

## Abstract

The *Inocybe tiliae* group (sect. *Marginatae*) was previously known mainly from Europe. In this study, a multigene phylogeny of the group was reconstructed based on ITS, LSU, and *rpb2* sequences, incorporating extensive collections from subtropical and temperate China for the first time. Four new species, *I. qiongzhuensis***sp. nov**., *I. ravida***sp. nov**., *I. xihuensis***sp. nov**., and *I. zijinshanensis***sp. nov**., are described, and three taxa, *I. tiliae*, *I. dunensis*, and the *I. pallida*/*I. nobilis* complex, are reported for the first time from China. Phylogenetic analyses recovered a fully supported major clade comprising the former core lineage, *I. robertii*, the Dunensis clade, and the four new Chinese species. The Pseudohiulca clade is sister to this major clade, whereas the Velata clade is sister to all the aforementioned lineages. Based on these results, the circumscription of the Tiliae group is expanded to include the major clade, the Pseudohiulca clade, and the Velata clade, comprising 12 species plus the *I. pallida*/*I. nobilis* complex and the *I. pseudohiulca* complex. The broad Tiliae group is defined by a combination of morphological traits: robust basidiomata, a yellowish-ochraceous to dark brown pileus, a stipe often with pinkish tones, a bulbous or marginate stipe base, a stipe that is either entirely pruinose or at least pruinose in the upper half, and often a spermatic odor.

## Introduction

The genus *Inocybe* (Fr.) Fr. (*Inocybaceae, Agaricales*) comprises approximately 1,100 species worldwide, with approximately 150 species currently documented from China (Matheny et al. 2020; Chen et al. 2024; Hyde et al. 2024; Gao et al. 2025; Zhu et al. 2025; Li et al. 2026). Species of the genus are very important because many of them are poisonous mushrooms, and *I. rimosa* (Bull.) Kalchbr. (current name: *Pseudosperma rimosum* (Bull.) Matheny & Esteve-Rav.) is considered a medicinal fungus in China (Dai and Yang 2008; Wu et al. 2019, 2026).

Species of *Inocybe* sect. *Marginatae* Kühner are characterized by nodulose to nodulose-angular spores, a pruinose stipe (at least in the upper part) covered with caulocystidia, and typically a marginate bulb (Kühner 1933; Bon 1998). Within this section, Bon (1998) proposed subsect. *Oblectabiles* Bon to accommodate species with pinkish to reddish stipe tones. However, molecular phylogenetic studies have shown that morphological convergence is common in this group, and the traditional subsectional limits are not always monophyletic (Matheny et al. 2002; Matheny 2005; Ryberg et al. 2010).

Recent integrative studies have clarified species boundaries in a clade provisionally named the Tiliae group (Franchi et al. 2016; Esteve-Raventós et al. 2022). This clade belongs to sect. *Marginatae* and includes robust species with large, heterodiametric spores (average length >10 µm) and a stipe often with pinkish to reddish tints. Franchi et al. (2016) described *I. tiliae* Franchi, M. Marchetti & Papetti from urban Brescia, Italy, under *Tilia* trees. It differs from morphologically similar species such as *I. nobilis* (R. Heim) Alessio, *I. decipiens* Bres., and *I. oblectabilis* (Britzelm.) Sacc. by its subcylindrical to narrowly fusiform cystidia and distinctly knobby spores. In the phylogeny of Franchi et al. (2016), *I. tiliae* falls in a well-supported clade (Clade B) within sect. *Marginatae*, separate from the whitish-stiped Praetervisae clade (Clade A).

Subsequently, Esteve-Raventós et al. (2022) typified *I. oblectabilis* f. *macrospora* Kühner (≡ *I. robertii* Esteve-Rav. & A. Caball.) and re-examined the phylogeny of the Tiliae group. They showed that the Tiliae clade includes at least four European taxa: *I. tiliae*, *I. robertii*, *I. tenuiorparietalis* (E. Ludw.) E. Larss., Esteve-Rav. & Pancorbo (≡ *I. piceae* var. tenuiorparietalis E. Ludw.), and the *I. pallida*/*I. nobilis* complex. Based on ITS sequences from the holotype, *I. pachycaulis* E. Ludw. was synonymized with *I. tiliae*. In addition, *I. piceae* Stangl & Schwöbel, *I. pseudohiulca* Kühner, and *I. kohistanensis* Jabeen, I. Ahmad & Khalid form a separate unresolved complex (the Pseudohiulca clade), which was shown to be distinct from *I. robertii* (Jabeen et al. 2016; Esteve-Raventós et al. 2022).

In the same year, Bandini and colleagues described another new species, *I. sepiana* Bandini, Dondl & Dima, from calcareous, gravelly, or sandy habitats in central Europe (Bandini et al. 2022). It is characterized by small basidiomata, a blackish-brown pileus center with age, a tomentose to squamulose pileus surface, an entirely pruinose stipe with a blackish-brown rim at the base, and often subcapitate hymenial cystidia. In their ITS-LSU phylogeny (fig. 1 in Bandini et al. 2022), *I. sepiana* formed a strongly supported clade and was recovered as sister to a group containing *I. tiliae* and *I. nobilis sensu* Franchi et al.

Moreover, Bandini et al. (2022) included several other nodulose-spored species in their phylogenetic analyses that are closely related to the Tiliae clade. *Inocybe javorkae* Babos & Stangl, a sand-dwelling species with large oblong spores and a blackish stipe base (Babos and Stangl 1985), was resolved as sister to *I. dunensis* P.D. Orton with high support. *Inocybe dunensis*, a psammophilous species with a whitish velipellis and oblong-nodulose spores (Orton 1960), clustered together with *I. javorkae* and *I. velata* Franchi & M. Marchetti. *Inocybe velata*, originally described from Mediterranean coastal pine forests, formed a fully supported clade sister to what the authors considered *I. decipiens*. *Inocybe favrei* Bon, a boreal-alpine species, was discussed morphologically as close to *I. sepiana*, although earlier studies had already placed it within the *I. praetervisa* clade (Larsson et al. 2018). These results indicate that the Tiliae group and its allied taxa are phylogenetically more diverse than previously thought, comprising both habitat generalists and specialists adapted to sandy, calcareous, or alpine environments.

Despite these advances, the phylogenetic boundaries of the Tiliae group and its relationships to morphologically similar nodulose-spored lineages remain unclear. Most studies have concentrated on European taxa, and the diversity of the group in other regions (particularly East Asia) remains largely unexplored. Preliminary evidence, including unpublished sequences and environmental samples, suggests the presence of morphologically similar taxa in subtropical and temperate Asia, but their phylogenetic affinities and species boundaries have not been critically examined. Therefore, a combined morphological and molecular approach is needed to reconstruct a robust phylogeny of the Tiliae group, to clarify its species diversity in China, and to reassess its morphological circumscription.

## Materials and methods

### Sampling and morphological examination

Basidiomata investigated in the present study were collected from Jilin, Qinghai, Shanghai, Zhejiang, Jiangsu and Yunnan, China. Macromorphological characters were documented from fresh basidiomata in the field, and color notations were determined following Kornerup and Wanscher (1978). Habitat details, including vegetation type, substrate, and associated plant species, were recorded at each locality. Geographic coordinates and elevation were obtained using a handheld GPS device. Specimens were dehydrated in an electric oven at 45 °C and subsequently stored in sealed plastic bags with silica gel (Yu et al. 2020). Voucher specimens were deposited in the Herbarium of the Changbai Mountain Nature Reserve (ANTU) with FCAS numbers.

For microscopic observation, hand-cut sections of the pileipellis, stipitipellis, lamellae, and context tissues were mounted in 5% KOH solution and examined under an Olympus CX23 compound microscope. Micromorphological structures, including basidiospores, basidia, cheilocystidia, pleurocystidia, caulocystidia, pileipellis, and stipitipellis, were photographed and measured. All measurements were taken from mounted sections in KOH. Basidiospore dimensions exclude the apiculus; basidial measurements exclude sterigmata.

Spore measurements are presented as [*n/m/p*], where *n* denotes the total number of spores measured, *m* the number of basidiomata examined, and *p* the number of collections (Ge et al. 2021; Liu et al. 2021). Spore size ranges are expressed as (*A*) *c–d* (*B*), where *A* and *B* represent the extreme values, and *c* and *d* indicate the 5^th^ and 95^th^ percentiles, respectively. The quotient *Q* refers to the length/width ratio of a single spore, with *Qm* representing the mean value of all *Q* measurements.

### DNA extraction, PCR amplification, and sequencing

Total genomic DNA was extracted from dried herbarium material using the NuClean Plant Genomic DNA Kit (CWBIO, Beijing, China) following the manufacturer’s instructions. Three nuclear loci were targeted for amplification: the internal transcribed spacer (ITS), the large subunit (LSU), and the protein-coding gene *rpb2*.

Polymerase chain reaction (PCR) amplifications were performed in a total volume of 25 µL, containing 12.5 µL of 2× Taq Plus MasterMix (including dye), 1 µL of each forward and reverse primer (10 µM), 1 µL of template DNA, and 9.5 µL of ddH_2_O. Primer pairs used for amplification were ITS1F/ITS4 for ITS (White et al. 1990; Gardes and Bruns 1993), LR0R/LR7 for LSU (Vilgalys and Hester 1990), and bRPB2-6F/bRPB2-7.1R for *rpb2* (Matheny 2005). For ITS and LSU, the thermal cycling profile comprised an initial denaturation at 94 °C for 1 min, followed by 35 cycles of 95 °C for 30 s, 52 °C for 1 min, and 72 °C for 1 min, with a final extension at 72 °C for 10 min. Amplification of *rpb2* followed a similar regime but with an annealing temperature of 50 °C and a final extension at 72 °C for 8 min. All PCR products were examined on 1% agarose gels and sent to Sangon Biotech (Shanghai, China) for purification and Sanger sequencing in both directions.

### Phylogenetic analyses

The dataset comprised sequences generated from the collections in the present study and publicly available sequences retrieved from GenBank, with a focus on previously described species of sect. *Marginatae* and related lineages (Horak et al. 2015; Franchi et al. 2016; Larsson et al. 2018; Esteve-Raventós et al. 2022; Bandini et al. 2022; Matheny et al. 2022). Sequences were aligned using the MAFFT online server (https://mafft.cbrc.jp/alignment/server/; Katoh et al. 2019) under the E-INS-i strategy, and the resulting alignments were manually refined in BioEdit (Hall 1999). The three gene fragments (ITS, LSU, and *rpb2*) were concatenated in MEGA v5.0 (Tamura et al. 2011). *Nothocybe distincta* (K.P.D. Latha & Manim.) Matheny & K.P.D. Latha was selected as the outgroup.

Maximum likelihood (ML) inference was performed using IQ-TREE (Trifinopoulos et al. 2016), employing the automatic model selection function and 1000 ultrafast bootstrap replicates. Bayesian inference (BI) was conducted in MrBayes v3.2.7a (Ronquist et al. 2012), with the best-fit substitution model for each gene partition determined by MrMTgui 1.0 (Swofford 2002). Four independent Markov chain Monte Carlo (MCMC) chains were run for 10 million generations, sampling trees every 100 generations. Convergence was assessed by the average standard deviation of split frequencies (< 0.01), and the first 25% of sampled trees were discarded as burn-in. Posterior probabilities (PP) were calculated from the remaining trees.

## Results

### Molecular phylogeny

A total of 86 samples, including 24 newly sequenced collections and 60 newly obtained sequences from the present study (Suppl. material 1), were analyzed. The final dataset comprised three concatenated gene fragments with a total length of 2,691 bp: ITS (727 bp), *rpb2* (680 bp), and LSU (1,284 bp). For ML inference, IQ-TREE was used, with ModelFinder selecting TRN+I+G for both *rpb2* and LSU and TVM+I+G for ITS; the ML tree had a log-likelihood score of –14376.430. For BI, MrBayes was run with the GTR+I+G model chosen by MrModeltest. The MCMC analysis was carried out for 340,000 generations, with trees sampled every 100 generations, and burn-in was set to 25%. Convergence was achieved with a split-frequency standard deviation of 0.009765. The effective sample size (ESS) of 1045.80 and potential scale reduction factor (PSRF) of 1.003 confirmed the adequacy of sampling and convergence.

**Figure 1.**
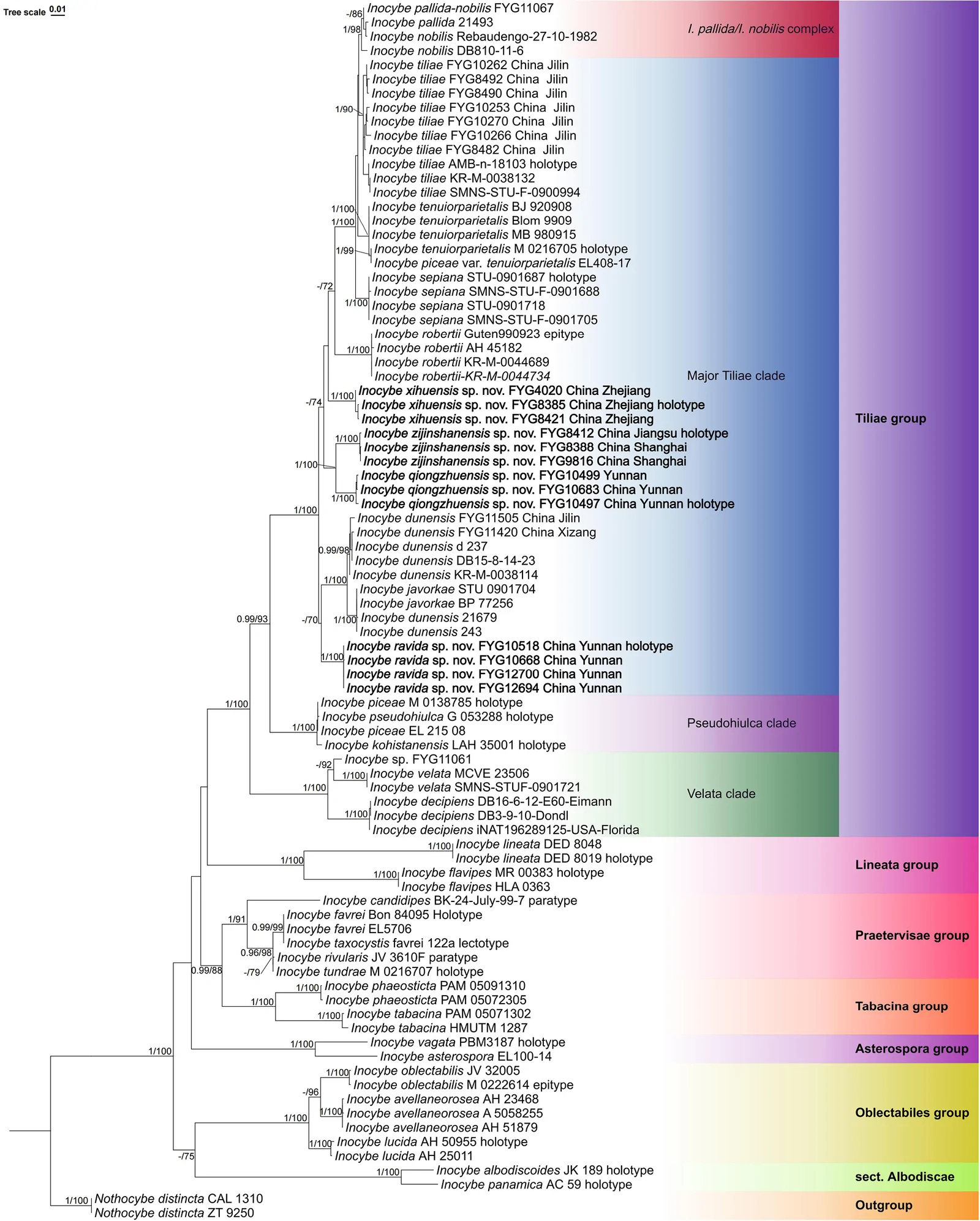
Maximum likelihood phylogeny of the Tiliae group and related lineages in *Inocybe* sect. *Marginatae* based on ITS, LSU, and *rpb2* sequences. Bootstrap values (≥ 70%) and Bayesian posterior probabilities (≥ 0.95) are shown near branches. The scale bar indicates substitutions per site. New species are in bold.

ML and BI analyses yielded largely congruent topologies; only the ML tree is presented in Fig. 1. The ingroup taxa were resolved into seven well-supported major clades: the *Tabacina* group (PP/BS = 1.00/100), the Praetervisae group (1.00/91), the Oblectabilis group (1.00/100), sect. *Albodiscae* (1.00/100), the Asterospora clade (1.00/100), the Lineata clade (1.00/100), and the Tiliae clade together with its allied lineages. Within the Praetervisae clade, *I. favrei* was recovered in a subclade with *I. rivularis* and *I. tundrae*. With the exception of a poorly supported group uniting sect. *Albodiscae* and the Oblectabilis clade (PP/BS = –/75) and a well-supported group comprising the Praetervisae and Tabacina groups (0.99/88), the phylogenetic relationships among these nodulose-spored lineages remained largely unresolved, reflecting the complex evolutionary history of this assemblage.

The four putative new species were each recovered as independent, fully supported lineages. *Inocybe xihuensis* clustered with the clade comprising *I. tiliae*, *I. tenuiorparietalis*, the *I. pallida*/*I. nobilis* complex, *I. sepiana*, and *I. robertii*, but with limited support (PP/BS = –/67). *Inocybe zijinshanensis* and *I. qiongzhuensis* formed a strongly supported sister pair (PP/BS = 1.00/100), a relationship consistent with their similar basidiomata and pigmentation. Together with *I. xihuensis*, these two species and the former core lineage constituted a poorly supported clade (PP/BS = –/74). This clade, in turn, formed a well-supported group (PP/BS = 1.00/100) together with *I. ravida* and the Dunensis clade (*I. dunensis* and *I. javorkae*), comprising 10 species in total and provisionally referred to here as the “major Tiliae clade” (see Discussion).

Seven collections from poplar plantations in northeastern China (FYG8482, FYG8490, FYG8492, FYG10253, FYG10262, FYG10266, and FYG10270) clustered with European material of *I. tiliae* with strong support (PP/BS = 1.00/90). Within this lineage, the Chinese collections exhibited some genetic differentiation from the European specimens, suggesting possible infraspecific variation or geographical structuring. Specimen FYG11067 from Qinghai was recovered within the *I. pallida*/*I. nobilis* complex (PP/BS = 1.00/98). Two specimens from Jilin (FYG11420) and Xizang (FYG11505) formed a strongly supported clade with European *I. dunensis* (PP/BS = 0.99/98). These results confirm the occurrence of *I. tiliae*, the *I. pallida*/*I. nobilis* complex, and *I. dunensis* in China.

The Pseudohiulca clade, corresponding to the *I. pseudohiulca* complex, received full support (PP/BS = 1.00/100) and was recovered as sister to the major clade. The Velata clade (*I. velata* and *I. decipiens sensu* Bandini), in turn, was placed as sister to all the aforementioned lineages. Within this clade, *I. velata* and *I. decipiens sensu* Bandini are noteworthy for their reduced stipe pruinescence, which is restricted to the apex or upper portion of the stipe. Specimen FYG11061 (FCAS4464), collected from subalpine shrublands in Gansu, China, was resolved as sister to *I. velata*. Although only a single overmature specimen was available, FYG11061 was observed to have a dark brown pileus and a robust stipe with brownish to pinkish tones, characters largely consistent with those of other taxa in the Tiliae group.

### Taxonomy

Based on combined morphological, ecological, and multilocus phylogenetic evidence, four new species and three newly recorded taxa of the Tiliae group are confirmed from China. The phylogenetic framework presented here, together with morphological data, supports an expanded circumscription of the Tiliae group to include the Pseudohiulca clade and the Velata clade.

#### Inocybe qiongzhuensis

Taxon classificationFungiAgaricalesInocybaceae

W.J. Yu & Y.G. Fan sp. nov.

4D0D8F0F-8DD1-548A-8577-A7D1AFE70B2D

864270

Figs 2, 3

##### Chinese name.

筇竹丝盖伞 (qióng zhú sī gài sǎn).

##### Etymology.

Named after Qiongzhu Temple, the type locality in Kunming, Yunnan Province, China.

##### Diagnosis.

The new species is characterized by a grayish-brown pileus, a stipe with pinkish tinge, entirely pruinose stipe surface, nodulose basidiospores, and hymenial cystidia with walls gradually thickening from base to apex. Most similar to *I. zijinshanensis*, but differs from it by the more prominently nodulose in basidiospores, longer and subutriform hymenial cystidia, and the cystidia often bearing a short pedicel.

##### Holotype.

China • Yunnan Province: Kunming City, Wuhua District, Qiongzhu Temple, 24 July 2024, in fagaceous forests, leg. Y.G. Fan & W.J. Yu, FYG10497 (FCAS4459).

##### Description.

***Basidiomata*** small. Pileus 10–20 mm diam., conical when young, then campanulate, applanate-convex at maturity, with a distinct umbilicate umbo at center; margin slightly incurved when young, initially appressed to stipe; surface dry, nearly smooth at disc, occasionally with uplifted squamules, at times with residual filamentous veil remnants at center when young, becoming radially cracked towards margin with age; dark brown (5F5) to brown (5D5) at center, gradually paler outwards, mostly pale brown (5E6) to pale brownish-yellow (4C4), pale yellowish-white (2A3) at margin. ***Lamellae*** adnexed, moderately crowded, 2–3 mm wide, unequal in length, alternately distributed with 3–4 tiers of lamellulae, edge indistinctly serrulate, paler; initially whitish (1A1) to yellowish-white (2A3), pale brownish-yellow (4C4) at maturity. ***Stipe*** 20–40 mm long, 3–5 mm wide, cylindrical, slightly curved, solid; base slightly swollen, up to 5 mm wide, submarginately bulbous; surface entirely pruinose; ivory white (1A1) when young, at maturity the outer surface gradually changing from pale yellowish-brown (5D5) at apex to ivory white (1A1) at base. ***Context*** fleshy in pileus, brown (5D5) at center, dark brown (5F5) at umbo, 0.5–2 mm thick; in stipe fibrillose, pale yellowish-brown (5D5) near stipe surface, ivory white (1A1) to grayish-white (1B2) internally and at base, gradually changing from ivory white (1A1) at apex to pale yellowish-brown (5D5) at base. ***Odor*** spermatic.

**Figure 2.**
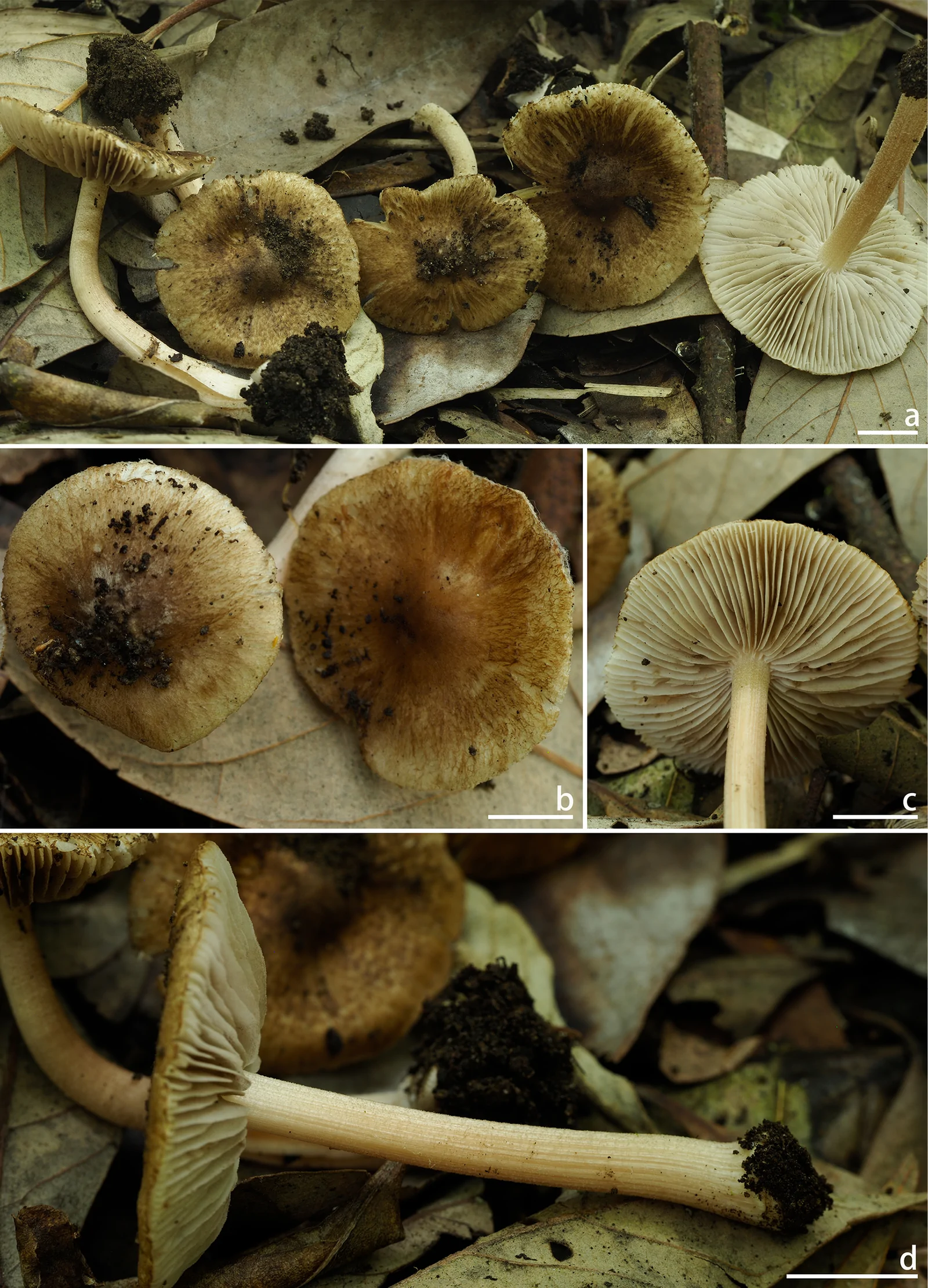
Basidiomata of *Inocybe qiongzhuensis* (FYG10497 (FCAS4459), holotype). **a–d**. Basidiomata *in situ*. Photos by Y.G. Fan. Scale bars: 10 mm (**a–d**).

**Figure 3.**
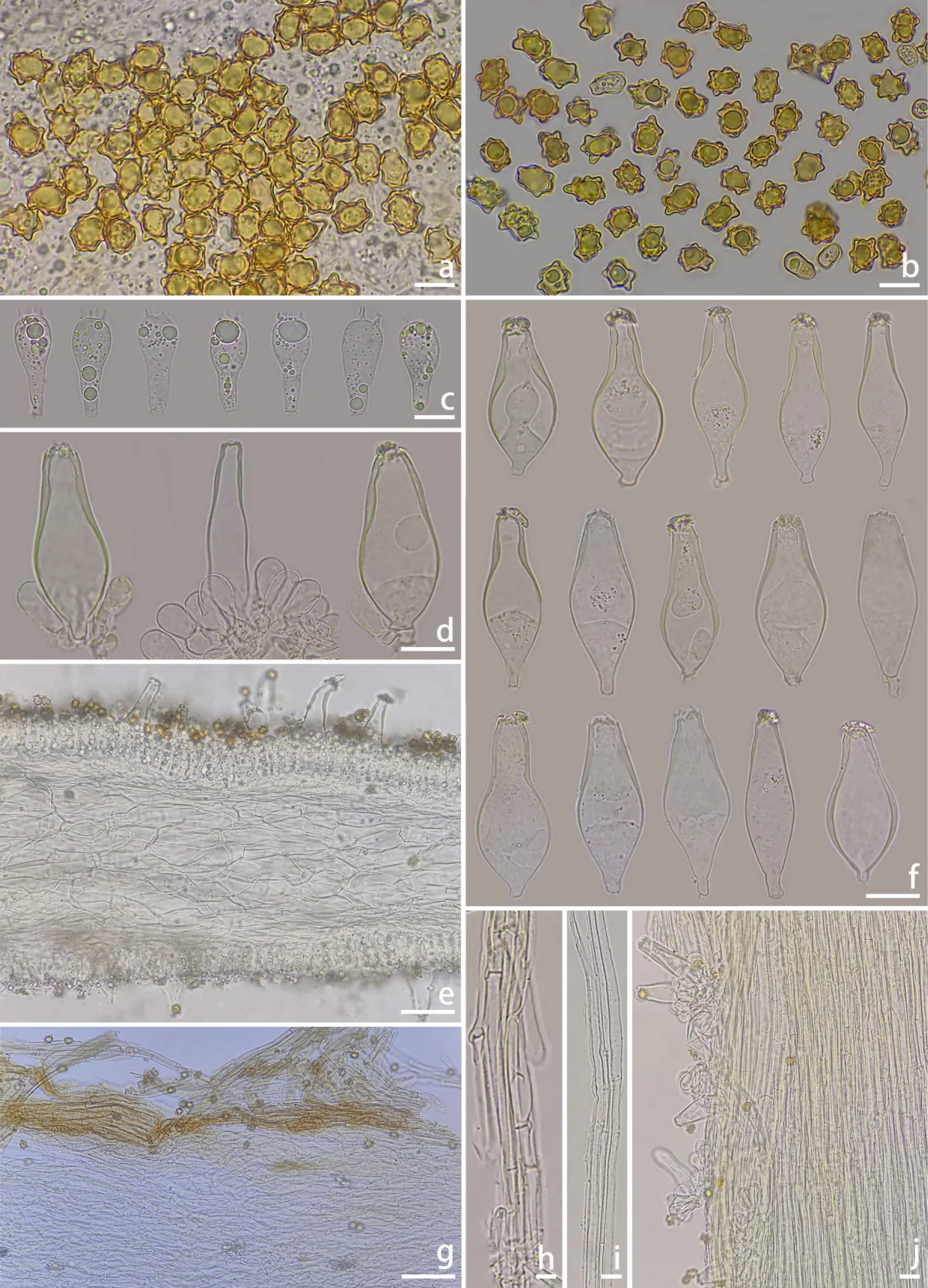
Microscopic features of *Inocybe qiongzhuensis* (FYG10497 (FCAS4459), holotype). **a, b**. Basidiospores; **c**. Basidia; **d**. Cheilocystidia and cheiloparacystidia; **e**. Cross-section of lamellae; **f**. Pleurocystidia; **g**. Pileipellis and pileal trama; **h**. Hyphae of stipitipellis; **i**. Hyphae of stipe trama; **j**. Caulocystidia and cauloparacystidia on stipe surface. Photos by Y. Li. Scale bars: 10 μm (**a–c, h–j**); 20 μm (**d–g**).

***Basidiospores*** [100/2/2] (8.2) 8.5–9.6–10.5 (11.1) × (6.3) 6.9–8.0–8.9 (9.2) μm, *Q* = (1.06) 1.12–1.21–1.42 (1.55), *Qm* ± SD = 1.21 ± 0.072, nodulose with 7–11 distinct, rounded nodules, yellow to yellowish-brown in 5% KOH, usually containing a large oily droplet. Basidia 29.8–36.4 × 9.9–12.7 μm, cylindrical to subclavate, with four sterigmata 3–4 μm long, thin-walled, colorless, with abundant contents, usually containing 1–2 oily droplets, occasionally transparent. ***Pleurocystidia*** 57–67–76 × 20–23–28 μm (*n* = 30), broadly fusiform to elongate-fusiform, occasionally subutriform, apex mostly obtuse, often encrusted with crystals, occasionally with very few contents, base gradually tapering to truncate, occasionally with a short pedicel, thick-walled, walls gradually thickening from base to apex, thickest at apex up to 3–4 μm, walls distinctly yellow, interior usually subhyaline, occasionally with very few contents. ***Cheilocystidia*** 44–54–64 × 20–22–26 μm (*n* = 30), similar to pleurocystidia, broadly fusiform to elongate-fusiform, often utriform, walls up to 2–3 μm thick at apex, distinctly yellow. Lamellae edge with abundant paracystidia, 15–20 × 5–9 μm, subclavate to inflated-clavate, thin-walled, colorless, transparent, without contents. ***Caulocystidia*** 43–73 × 17–23 μm, densely clustered on upper stipe, extending to base, gradually decreasing downwards, narrowly elongate-fusiform or subclavate, occasionally utriform, apex obtuse, smooth, without crystals or contents; thick-walled, walls gradually thickening from base to apex, up to 2–3 μm thick at apex. ***Cauloparacystidia*** 19–23 × 8–13 μm, abundant, subglobose, clavate or subcylindrical, thin-walled, colorless, transparent, without contents. ***Hymenophoral trama*** 83–120 μm wide, subregular, composed of inflated hyphae 5–19 μm diam. ***Pileipellis*** a cutis, 45–112 μm thick, distinctly yellow, composed of cylindrical hyphae 8–12 μm wide, thin-walled, pale yellow, transparent, surface rough, occasionally smooth. ***Pileal trama*** subregular, composed of elongate hyphae gradually becoming inflated downwards, 5–17 μm diam., smooth, thin-walled, colorless, transparent. ***Stipitipellis*** regularly arranged, hyphae cylindrical, 6–11 μm diam., smooth, pale yellow. ***Stipe trama*** regularly arranged, hyphae elongate-cylindrical, 5–14 μm diam., thin-walled, smooth, colorless, transparent. ***Oleiferous hyphae*** common in hymenium and pileipellis, less frequent in pileal trama, 4–10 μm wide, with tuberculate nodules, pale yellow to yellow. ***Clamp connections*** present in all tissues.

##### Habitat.

In subtropical evergreen broad-leaved forests dominated by *Fagaceae*.

##### Geographical distribution.

Known only from Yunnan Province, China.

##### Additional specimens examined.

China • Yunnan Province: Kunming City, Wuhua District, Heilinpu Subdistrict, Yu’an Mountain, Heiqiong Road, Qiongzhu Temple, 24 July 2024, in fagaceous forests, leg. Y.G. Fan & W.J. Yu, FYG10499 (FCAS4457); same locality, 1 August 2024, in fagaceous forests, leg. Y.G. Fan & W.J. Yu, FYG10683 (FCAS4458).

##### Remarks.

This species is currently known only from its type locality, with basidiomata occurring from late July to early August. It is characterized by a grayish-brown pileus with appressed to slightly uplifted fibrils, a stipe surface with a pale flesh-pink tinge, distinctly nodulose basidiospores, and subutriform hymenial cystidia. In the three-gene phylogenetic tree, this species is sister to *I. zijinshanensis*. They share similar pileus and stipe morphology; microscopically, the basidiospore sizes of the two species are similar. However, *I. zijinshanensis* has more numerous nodules on the spores, broadly fusiform and shorter hymenial cystidia, and usually lacks a pedicel. North temperate taxa, such as *I. tiliae* and *I. sepiana*, are also phylogenetically closely related. However, *I. tiliae* has larger basidiomata, a more pink-tinged and more robust stipe, larger basidiospores, shorter and slenderer hymenial cystidia, and grows under *Tilia* or *Quercus* in urban or natural forests (Franchi et al. 2016), whereas *I. sepiana* has rather small basidiomata, a dark-brown pileus, less nodulose basidiospores, thicker-walled hymenial cystidia, and occurs in *Pinus* or *Salix* forests (Bandini et al. 2022).

#### Inocybe ravida

Taxon classificationFungiAgaricalesInocybaceae

W.J. Yu & Y.G. Fan sp. nov.

223D98A3-0517-5EB2-905C-7E3D5996F981

864273

Figs 4, 5

##### Chinese name.

灰黄丝盖伞 (huī huáng sī gài sǎn).

##### Etymology.

Referring to the grayish-yellow color of the pileus.

##### Diagnosis.

Basidiomata medium-sized; pileus earthy-yellow to ginger-yellow, sometimes grayish-yellow; stipe surface entirely pruinose; basidiospores with 7–11 small nodules; hymenial cystidia fusiform. Morphologically most similar to *I. qiongzhuensis*, but differs from it by the more yellow pileus, relatively larger basidiospores, and smaller, fusiform hymenial cystidia.

##### Holotype.

China • Yunnan Province: Kunming City, Wuhua District, Heilinpu Subdistrict, Yu’an Mountain, Qiongzhu Temple, in fagaceous forests, 24 July 2024, leg. Y.G. Fan & W.J. Yu, FYG10518 (FCAS4462).

##### Description.

***Basidiomata*** medium-sized. Pileus 20–32 mm diam., conical when young, then campanulate, applanate-convex at maturity, with a distinct umbilicate umbo at center; margin slightly incurved when young, expanding at maturity, uplifted when old; surface dry, without uplifted squamules, nearly smooth at disc, occasionally with very few residual filamentous veil remnants at center when young, becoming radially cracked from center to margin with age; warm yellow (4B8) to ginger-yellow (4A8) at center, sometimes grayish-brown (5C4–5C5), paler outwards, mostly earthy-yellow (4A4) to pale earthy-gray (4B4), pale yellowish-white (2B2) at margin. ***Lamellae*** adnexed, moderately crowded, 2–3 mm wide, unequal in length, alternately distributed with 3–4 tiers of lamellulae, edge indistinctly serrulate, paler; white (1A1) to grayish-white (3A2), with yellowish-brown (3C2) tinge at maturity, occasionally with small brown spots on lamellae. ***Stipe*** 30–40 mm long, 3–4 mm wide, cylindrical, slightly curved, solid; base slightly swollen, up to 7 mm wide, submarginately bulbous; entirely pruinose with distinct longitudinal striae; surface yellowish-white (5B3) when young, at maturity gradually changing from pale brownish-yellow (5C4) at apex to ivory white (2A1) at base. ***Context*** fleshy in pileus, white (1A1), dark brown (5E4) at umbo, 0.5–2 mm thick; in stipe fibrillose, pale brownish-yellow (5C4) near surface, ivory white (1A1) to yellowish-white (4C3) internally and at base. ***Odor*** spermatic.

**Figure 4.**
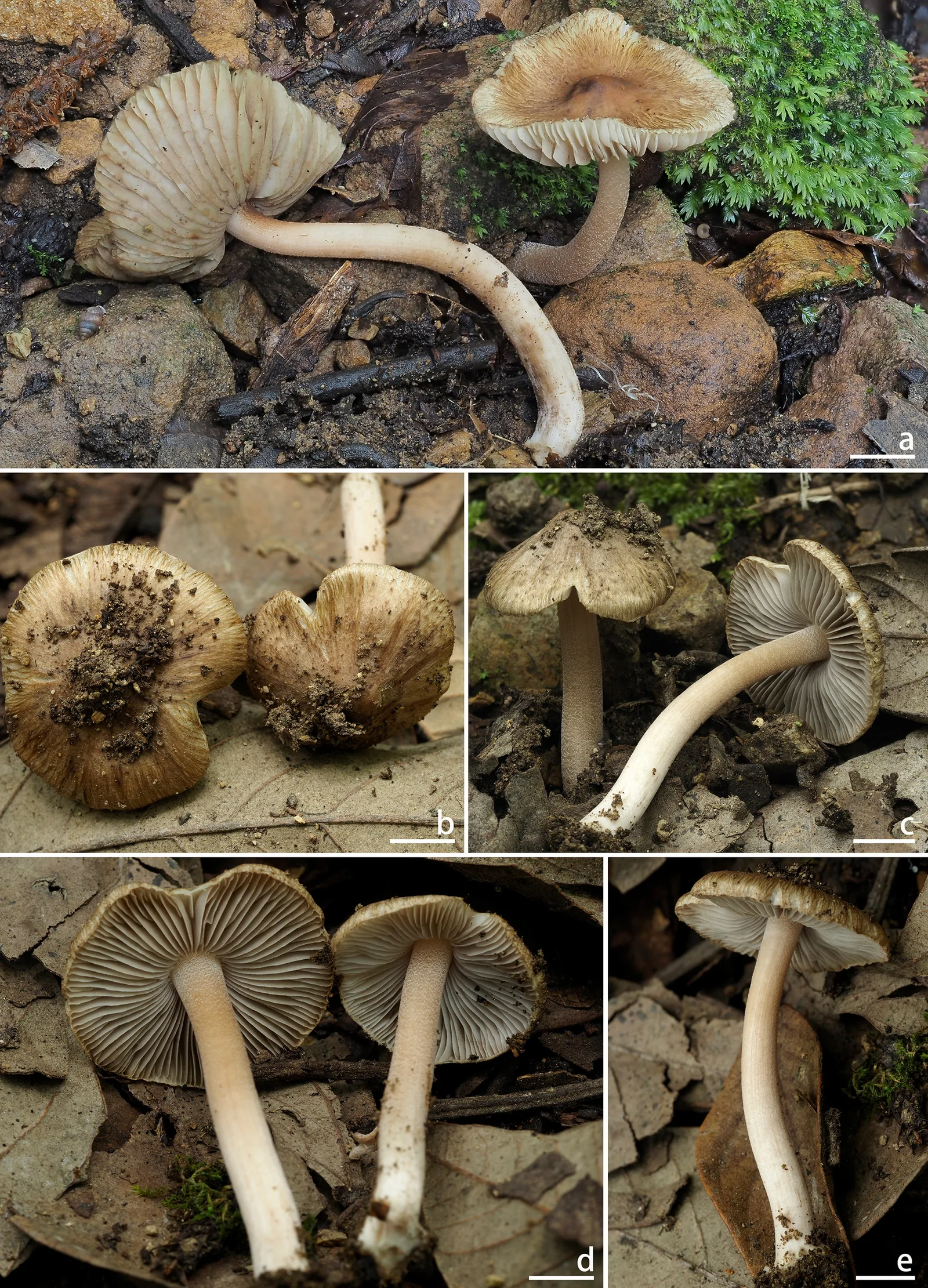
Basidiomata of *Inocybe ravida* (FYG10518 (FCAS4462), holotype). **a–e**. Basidiomata *in situ*. Photos by Y.G. Fan. Scale bars: 10 mm (**a–e**).

**Figure 5.**
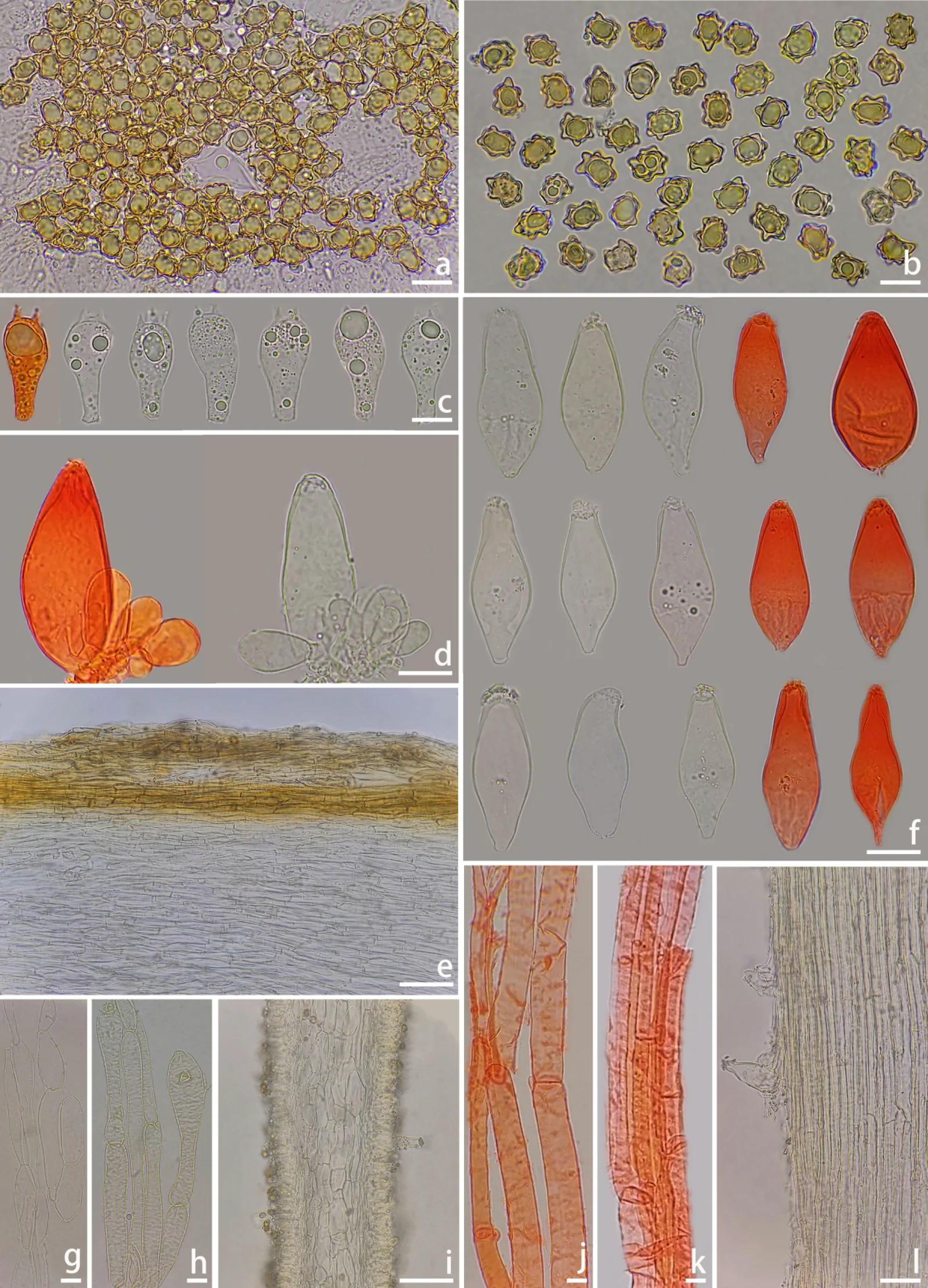
Microscopic features of *Inocybe ravida* (FYG10518 (FCAS4462), holotype). **a, b**. Basidiospores; **c**. Basidia; **d**. Cheilocystidia and cheiloparacystidia; **e**. Pileipellis and part of pileal trama; **f**. Pleurocystidia; **g**. Hyphae of pileal trama; **h**. Hyphae of pileipellis; **i**. Cross-section of lamellae; **j**. Hyphae of stipitipellis; **k**. Hyphae of stipe trama; **l**. Caulocystidia on stipe surface. Photos by Y. Li. Scale bars: 10 μm (**a–c, g, h, j–l**); 20 μm (**d–f, i**).

***Basidiospores*** [100/2/2] (9.1) 9.7–10.8–11.9 (12.9) × (7.5) 7.9–9.0–9.8 (10.1) μm, *Q* = 1.10–1.21–1.35 (1.45), *Qm* ± SD = 1.21 ± 0.068, nodulose, with 7–11 distinct rounded nodules, yellow to yellowish-brown in 5% KOH, usually containing a large oily droplet, occasionally two small oily droplets. Basidia 28.9–36.1 × 11.1–15.7 μm, subclavate, with four sterigmata 4–5 μm long, occasionally 2-spored, thin-walled, colorless, with abundant contents, usually containing 1–4 oily droplets, occasionally without contents. ***Pleurocystidia*** 60–66–70 × 19–25–30 μm (*n* = 30), narrowly elongate-fusiform, broadly fusiform, occasionally sublageniform, apex mostly obtuse, often encrusted with crystals, occasionally with very few contents, base gradually tapering to truncate, occasionally base rounded-tapering, occasionally with a short pedicel, thick-walled, walls gradually thickening from base to apex, thickest at apex up to 1–2 μm, occasionally up to 3 μm, walls distinctly yellow, interior usually subhyaline, occasionally with very few contents. ***Cheilocystidia*** 44–50–60 × 16–21–25 μm (*n* = 30), similar to pleurocystidia, subbroadly fusiform, walls up to 2–3 μm thick at apex, distinctly yellow. Lamellae edge with abundant paracystidia, 14–21 × 7–11 μm, subclavate to inflated-clavate, occasionally guttulate, thin-walled, colorless, transparent, without contents. ***Caulocystidia*** 35–58 × 18–31 μm, present over entire stipe surface (entirely pruinose), densely clustered on upper stipe, gradually decreasing downwards to base, broadly fusiform, apex obtuse, smooth, without crystals or contents; thick-walled, walls gradually thickening from base to apex, up to 2–3 μm thick at apex, distinctly yellow. ***Cauloparacystidia*** 20–30 × 13–20 μm, abundant, subglobose, clavate or subcylindrical, thin-walled, colorless, transparent, without contents. ***Hymenophoral trama*** 65–125 μm wide, subregular, composed of inflated hyphae 4–16 μm diam. ***Pileipellis*** a cutis, 76–164 μm thick, distinctly yellow, composed of cylindrical hyphae 8–15 μm wide, thin-walled, pale yellow, transparent; outermost hyphae of pileipellis rough, inflated, occasionally elongate; inner hyphae of pileipellis smooth, elongate. ***Pileal trama*** subregular, composed of elongate to inflated hyphae 6–18 μm diam., smooth, thin-walled, colorless, transparent. ***Stipitipellis*** regularly arranged, hyphae cylindrical, 5–13 μm diam., smooth, occasionally rough, pale yellow. ***Stipe trama*** regularly arranged, hyphae elongate-cylindrical, 4–13 μm diam., thin-walled, smooth, colorless, transparent. ***Oleiferous hyphae*** common in pileus and stipe, more abundant in stipe than in pileus, very sparse in hymenium, 6–10 μm wide, with tuberculate nodules, pale yellow to yellow. ***Clamp connections*** present in all tissues.

##### Habitat.

In subtropical evergreen broad-leaved forests dominated by *Fagaceae*.

##### Geographical distribution.

Known only from Yunnan Province, China.

##### Additional specimens examined.

China • Yunnan Province: Kunming City, Wuhua District, Heilinpu Subdistrict, Yu’an Mountain, Qiongzhu Temple, in fagaceous forests, 1 August 2024, leg. Y.G. Fan & W.J. Yu, FYG10668 (FCAS4463); same locality, 28 August 2025, leg. Y.G. Fan & W.J. Yu, FYG12694 (FCAS4465), FYG12700 (FCAS4466).

##### Remarks.

This species is currently known only from Kunming, Yunnan Province, with basidiomata occurring in August. It is characterized by a grayish-yellow pileus with appressed fibrils, a stipe surface covered with pruinose granules, basidiospores with 7–11 distinct nodules, and fusiform hymenial cystidia. Multigene phylogenetic analyses show that this species is closely related to *I. tiliae*, *I. qiongzhuensis*, and *I. javorkae*. However, *I. tiliae* has more robust basidiomata, an ochraceous-flesh-colored to yellowish-flesh-colored pileus, lageniform to subcylindrical hymenial cystidia, and grows mainly under *Tilia* (Franchi et al. 2016). *Inocybe qiongzhuensis* is similar in basidioma size, morphology, and habitat to *I. ravida*, but its pileus is duller, its basidiospores are relatively smaller, and its hymenial cystidia are subutriform and larger. *Inocybe javorkae* differs in having larger basidiomata, a squamulose pileus, a darker and larger stipe, angulate basidiospores that are almost non-nodulose, clavate to broadly clavate hymenial cystidia with attenuate bases, and walls up to 3 (4) μm thick (Babos and Stangl 1985; Bandini et al. 2022).

#### Inocybe zijinshanensis

Taxon classificationFungiAgaricalesInocybaceae

W.J. Yu, Y.G. Fan & X. Chen sp. nov.

88680E51-0504-5329-B364-417037441806

864276

Figs 6, 7

##### Chinese name.

紫金山丝盖伞 (zǐ jīn shān sī gài sǎn).

##### Etymology.

Named after Zijinshan (Purple Mountain), the type locality in Nanjing, Jiangsu Province, China, where this species was first collected.

##### Diagnosis.

Pileus covered with appressed, pale brown, small block-like squamules; stipe with pale flesh-pink tinge; basidiospores with 7–11 small nodules; hymenial cystidia broadly fusiform. Sister to *I. qiongzhuensis* from Yunnan, but differs in having more numerous but less prominent nodules on the basidiospores, shorter and broadly fusiform hymenial cystidia without a pedicel.

##### Holotype.

China • Jiangsu Province: Nanjing City, Xuanwu District, Zijinshan, in fagaceous forests, 15 July 2023, leg. X. Chen, Chen365/FYG8412 (FCAS4454).

##### Description.

***Basidiomata*** small. Pileus 20–30 mm diam., conical to hemispherical when young, then convex to applanate, with a distinct blunt umbo at center; margin incurved when young, expanding at maturity; surface moist, without uplifted squamules, disc smooth and shiny, with a few brown appressed fibrils at center, margin often cracked at maturity; dark brown (6E6) to pale brown (5D6) at center, gradually paler outwards, mostly pale brown (5B3) to pale brownish-yellow (4C3), margin yellowish-white (1A4). ***Lamellae*** adnexed, moderately crowded, 2–3 mm wide, unequal in length, with 2–3 tiers of lamellulae, edge indistinctly serrulate; ivory white (1A1) to pale yellow (4A5), occasionally with small black spots on lamellae. ***Stipe*** 35–55 mm long, 3–7 mm wide, cylindrical, slightly curved, solid; base slightly swollen, up to 10 mm wide, with a submarginate base; surface entirely covered with abundant white pruina; ivory white (1A1) with a pale flesh-pink tinge (5A2). ***Context*** fleshy in pileus, white (1A1) to dirty white (1B2), 0.5–2 mm thick; in stipe fibrillose, ivory white (1A1) with a pale flesh-pink tinge (5A2). ***Odor*** spermatic.

**Figure 6.**
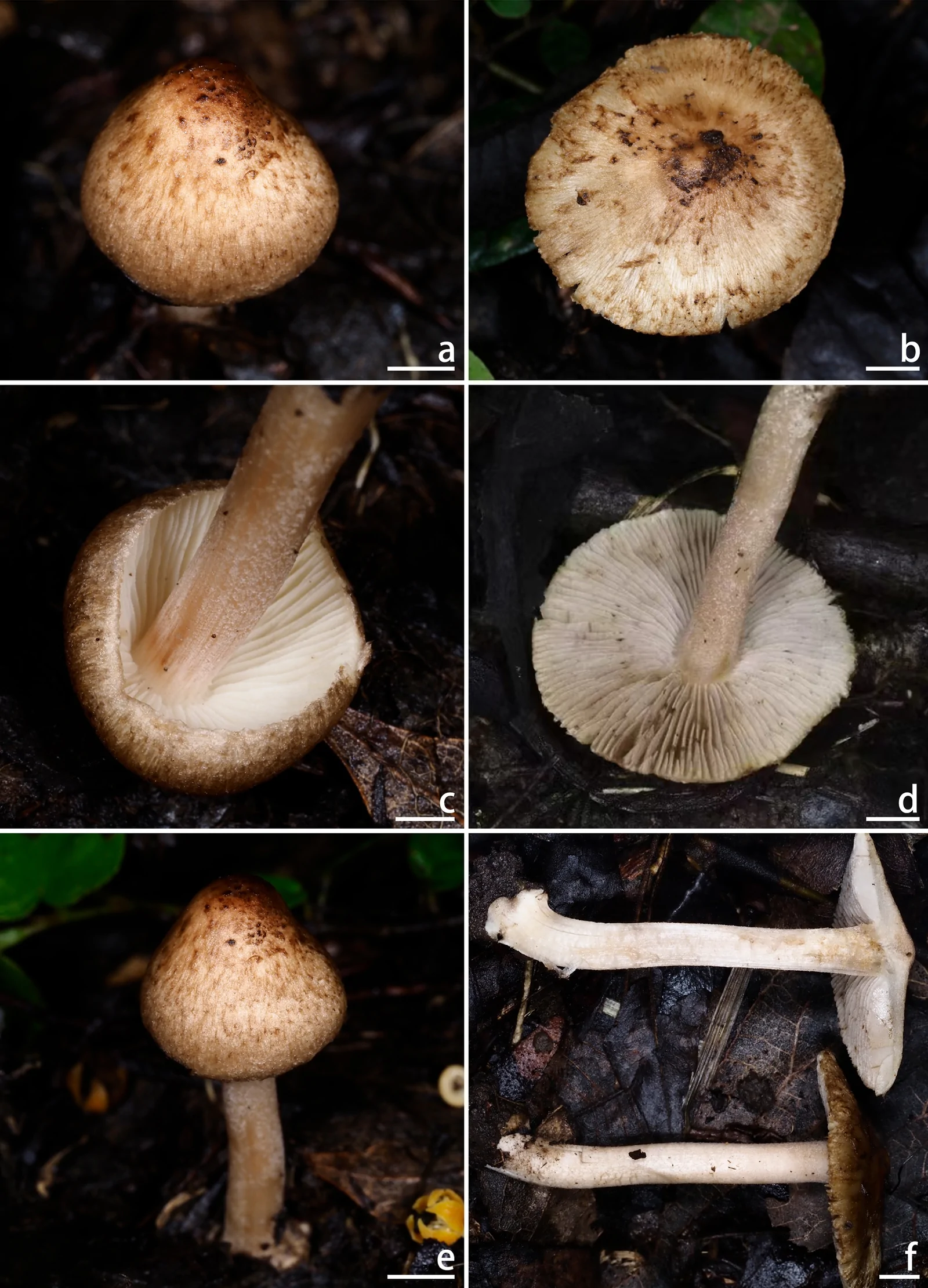
Basidiomata of *Inocybe zijinshanensis* (FYG8412 (FCAS4454), holotype). **a–f**. Basidiomata *in situ*. Photos by X. Chen. Scale bars: 5 mm (**a–f**).

**Figure 7.**
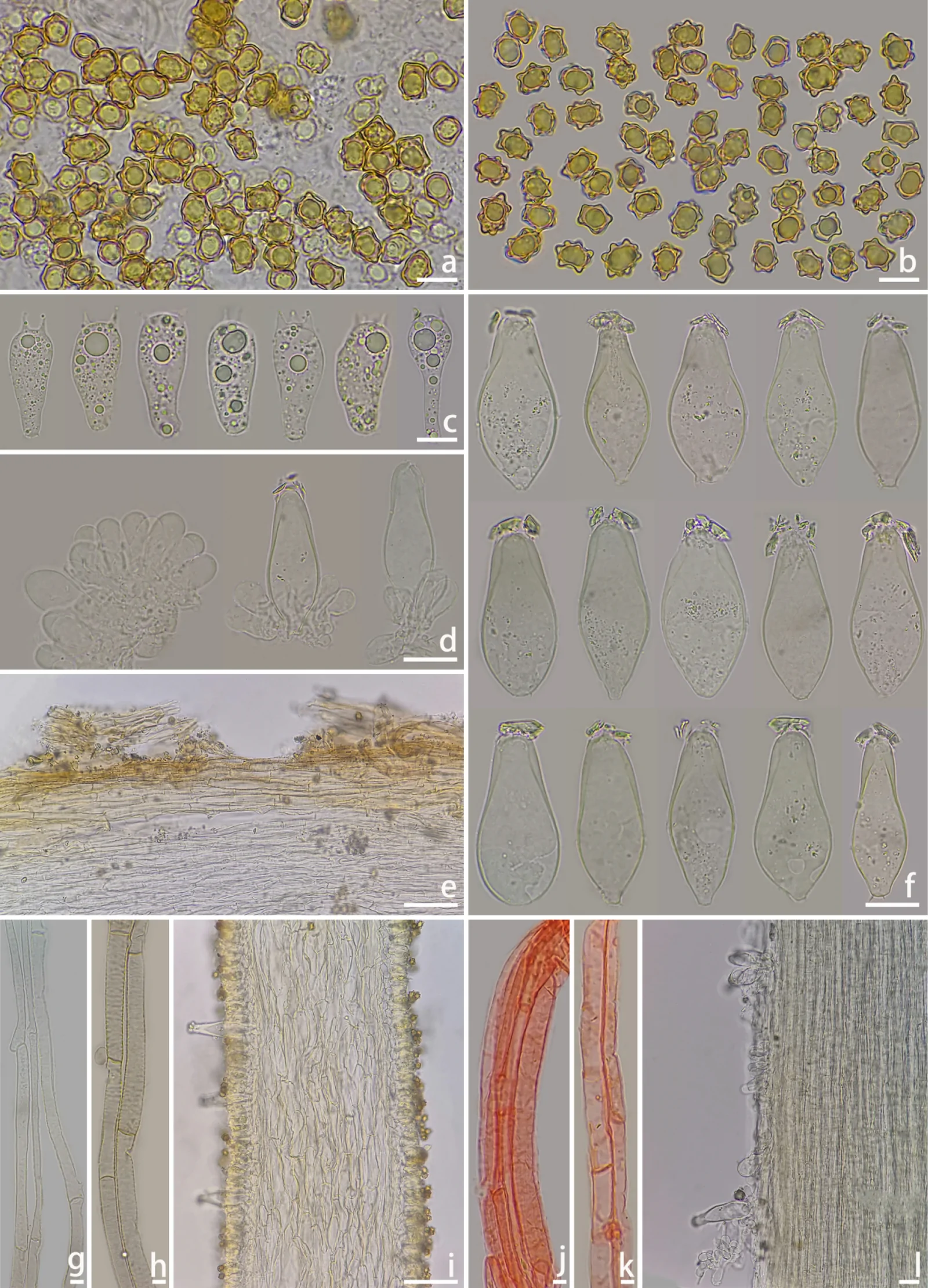
Microscopic features of *Inocybe zijinshanensis* (FYG8412 (FCAS4454), holotype). **a, b**. Basidiospores; **c**. Basidia; **d**. Cheilocystidia; **e**. Pileipellis; **f**. Pleurocystidia; **g–h**. Hyphae of pileipellis; **i**. Cross-section of lamellae; **j**. Hyphae of stipitipellis; **k**. Hyphae of stipe trama; **l**. Caulocystidia on stipe surface. Photos by Y. Li. Scale bars: 10 μm (**a–c, g, h, j–l**); 20 μm (**d–f, i**).

***Basidiospores*** [100/2/2] (7.9) 8.4–9.5–10.3 (10.9) × (5.8) 7.1–7.8–8.4 (8.8) μm, *Q* = 1.14–1.23–1.38, *Qm* ± SD = 1.23 ± 0.048, nodulose, with 7–11 distinct rounded nodules, yellow to yellowish-brown, usually containing oily droplets. Basidia 24–36 × 12–15 μm, broadly clavate to narrowly elongate-clavate, with four sterigmata 3–6 μm long, occasionally 2-spored, thin-walled, colorless, with abundant contents, usually containing 1–4 oily droplets. ***Pleurocystidia*** 48–55–63 × 18–23–25 μm (*n* = 30), broadly fusiform, narrowly elongate-fusiform, occasionally bulbous, apex often encrusted with crystals, occasionally with very few contents, base gradually tapering to truncate, occasionally rounded-tapering, occasionally with a short pedicel, thick-walled, walls gradually thickening from base to apex, thickest at apex up to 2–3 (4) μm, walls distinctly yellow, interior occasionally with very few contents. ***Cheilocystidia*** 46–51–55 × 16–20–23 μm (*n* = 30), similar to pleurocystidia, broadly fusiform, walls up to 2–3 μm thick at apex, distinctly yellow. Lamellae edge with abundant paracystidia, 16–23 × 7–13 μm, subclavate to inflated-clavate, thin-walled, colorless, transparent, without contents. ***Caulocystidia*** 37–76 × 18–25 μm, present over entire stipe surface, densely clustered on upper stipe, gradually decreasing downwards to base, narrowly elongate-fusiform, apex obtuse, smooth, often encrusted with crystals, occasionally without crystals, without contents, walls up to 2–3 μm thick at apex, subhyaline. Cauloparacystidia 17–25 × 6–15 μm, abundant, subglobose, clavate or subcylindrical, thin-walled, colorless, transparent, without contents. ***Hymenophoral trama*** 80–170 μm wide, subregular, composed of inflated hyphae 5–15 μm diam. ***Pileipellis*** a cutis, 92–138 μm thick, distinctly yellow, composed of cylindrical hyphae 5–11 μm wide, thin-walled, pale yellow, transparent, smooth, occasionally rough. ***Pileal trama*** subregular, composed of elongate hyphae gradually becoming inflated downwards; upper hyphae elongate, 7–14 μm wide; lower hyphae inflated, 17–27 μm wide, smooth, thin-walled, colorless, transparent. ***Stipitipellis*** regularly arranged, hyphae cylindrical, 5–10 μm diam., smooth, occasionally rough, pale yellow. ***Stipe trama*** regularly arranged, hyphae elongate-cylindrical, 5–9 μm diam., thin-walled, smooth, colorless, transparent. ***Oleiferous hyphae*** occasionally present in pileus and stipe, 4–10 μm wide, with tuberculate nodules, pale yellow to yellow. ***Clamp connections*** present in all tissues.

##### Habitat.

In subtropical evergreen broad-leaved forests dominated by *Fagaceae*.

##### Geographical distribution.

Known from Jiangsu and Shanghai, China.

##### Additional specimens examined.

China • Shanghai Municipality: Putuo District, Shandan Road green space, in fagaceous forests dominated by *Quercus texana* Buckley, 20 June 2023, leg. Y.K. Zhang, FYG8388 (FCAS4455); same locality, 1 November 2023, leg. Y.K. Zhang, FYG9816 (FCAS4456).

##### Remarks.

*Inocybe zijinshanensis* is currently known from subtropical eastern China, occurring under fagaceous trees from June to November. It is characterized by a pileus covered with appressed, pale brown, small block-like squamules, a pruinose stipe with a pale flesh-pink tinge, basidiospores with 7–11 small nodules, and broadly fusiform hymenial cystidia without a pedicel. Multigene phylogenetic analyses show that it is sister to *I. qiongzhuensis* from Yunnan Province. The latter differs in having more prominent nodules on the basidiospores and larger, subutriform hymenial cystidia that often bear a short pedicel. *Inocybe tiliae* is also closely related to the new species, but it has more robust basidiomata, a reddish tone in the pileus, a more pinkish stipe, larger basidiospores measuring (8.0) 9.0–11.0 (12.1) × (6.1) 6.8–8.1 (8.7) μm, slenderer hymenial cystidia, and occurs mainly in *Tilia* forests (Franchi et al. 2016).

#### Inocybe xihuensis

Taxon classificationFungiAgaricalesInocybaceae

W.J. Yu, Q.Q. Huang & J.M. Cai sp. nov.

12ECC4BB-F275-53B5-AD14-BC1406771B56

864277

Figs 8, 9

##### Chinese name.

西湖丝盖伞 (Xī Hú sī gài sǎn).

##### Etymology.

*xihuensis* refers to the type locality, West Lake (Xī Hú), Hangzhou, China.

##### Diagnosis.

The new species is characterised by a yellowish-brown pileus with a dark brown center, a stipe surface covered with granular pruina, basidiospores with 9–13 small rounded nodules, and hymenial cystidia obtusely fusiform to fusiform. Most similar to *I. qiongzhuensis*, but differs from it by the smaller and more numerous nodules on the basidiospores, and smaller hymenial cystidia.

##### Holotype.

China • Zhejiang Province: Hangzhou City, Xihu District, Hangzhou Botanical Garden, in fagaceous forests, 18 June 2023, leg. Q.Q. Huang, FYG8385 (FCAS4452).

##### Description.

***Basidiomata*** medium-sized. Pileus 22–35 mm diam., hemispherical when young, then plano-hemispherical, convex to applanate at maturity, with an indistinct obtuse umbo at center; margin incurved when young, expanding at maturity, without veil remnants; surface dry, with appressed fibrillose squamules, finely rimulose, margin cracked at maturity; dark brown (5E3–5E6) to grayish-brown (3C6) at center, gradually paler outwards, pale straw-yellow (3B7) to yellowish-brown (4C6), pale copper-yellow (4A5) at margin. ***Lamellae*** adnexed, moderately crowded, 2–3 mm wide, unequal in length, alternately distributed with 2–3 tiers of lamellulae, edge indistinctly serrulate, paler; white (1A1) to pale yellowish-white (1A2), becoming brownish (4B8) at maturity. ***Stipe*** 43–80 mm long, 4–8 mm wide, cylindrical, solid; base slightly swollen, up to 10 mm wide, marginately bulbous; upper part densely covered with white pruina, gradually sparser downwards but extending to base; white (1A1) to yellowish-white (1A2), with flesh-colored tinge (5A2). ***Context*** fleshy in pileus, dirty white (1B2), brownish (4D8) near pileipellis, 0.5–2 mm thick; in stipe fibrillose, pale yellowish-white (1A2) or with flesh-pink tinge (5A2) near surface. ***Odor*** spermatic.

**Figure 8.**
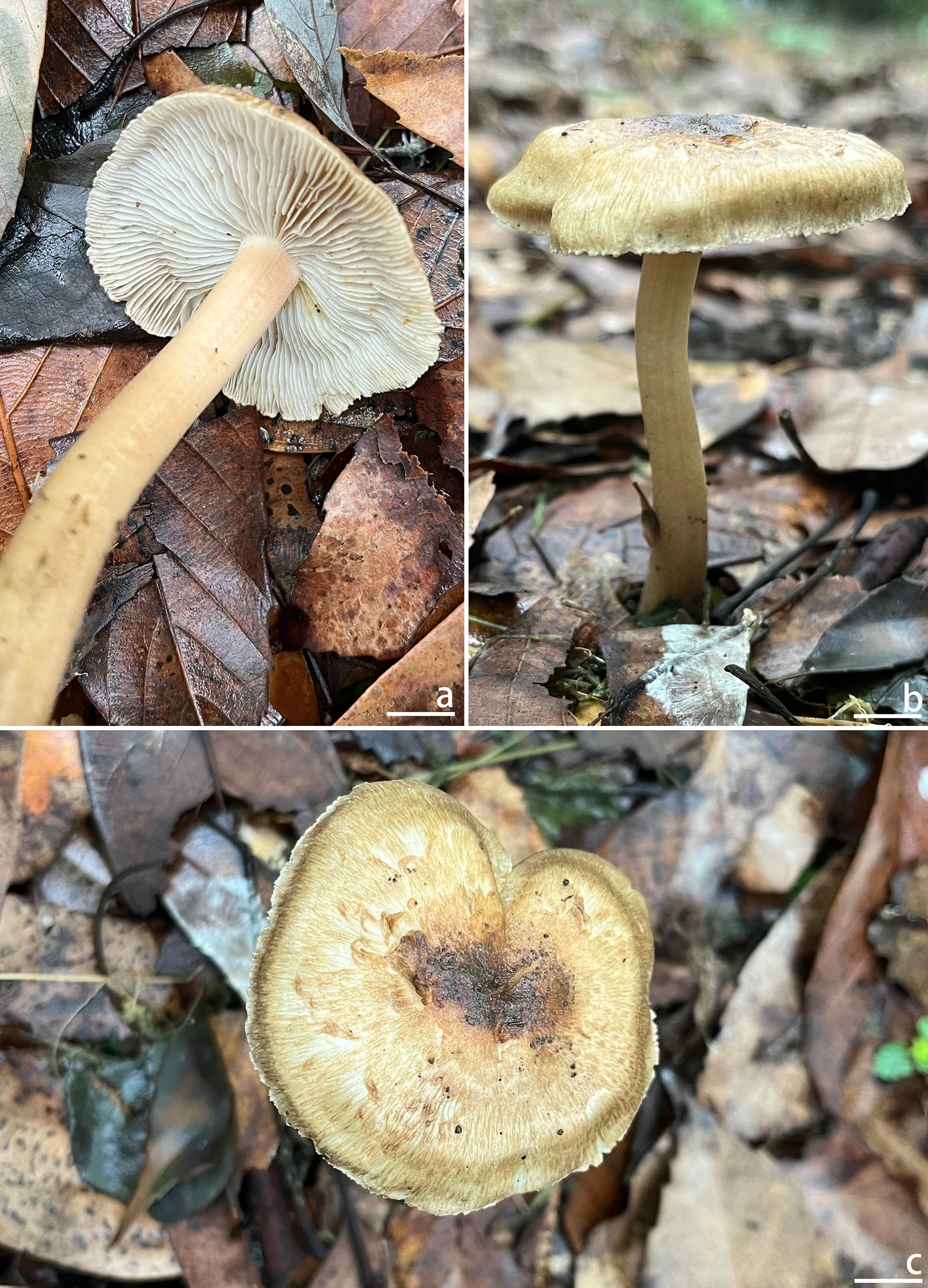
Basidiomata of *Inocybe xihuensis* (FYG8385 (FCAS4452), holotype). **a–c**. Basidiomata *in situ*. Photos by Q.Q. Huang. Scale bars: 10 mm (**a–c**).

**Figure 9.**
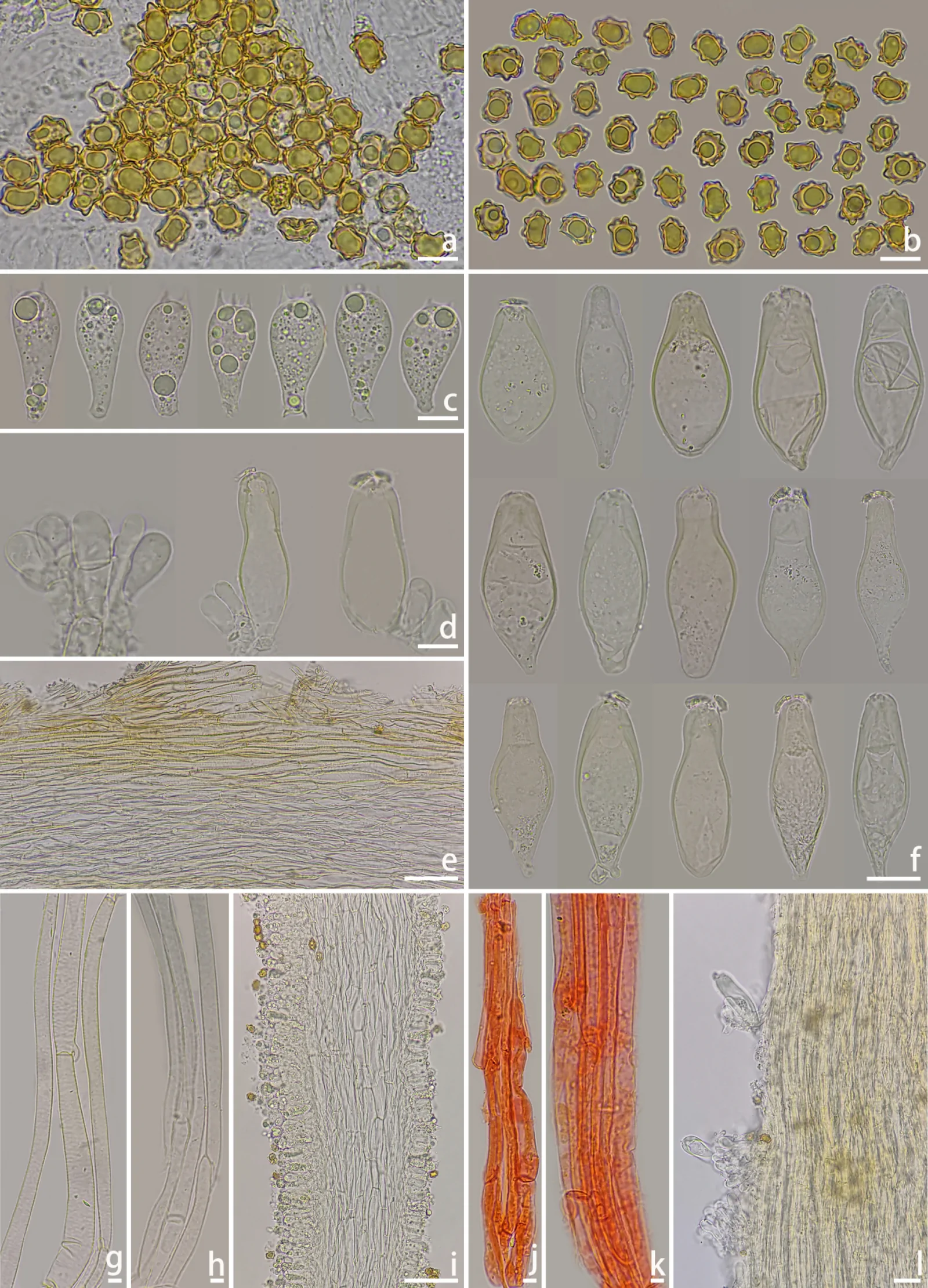
Microscopic features of *Inocybe xihuensis* (FYG8385 (FCAS4452), holotype). **a, b**. Basidiospores; **c**. Basidia; **d**. Cheilocystidia and cheiloparacystidia; **e**. Pileipellis and pileal trama; **f**. Pleurocystidia; **g, h**. Pileipellis hyphae; **i**. Cross-section of lamellae; **j**. Stipitipellis hyphae; **k**. Stipe trama hyphae; **l**. Caulocystidia on stipe surface. Photos by Y. Li. Scale bars: 10 μm (**a–c, g, h, j–l**); 20 μm (**d–f, i**).

***Basidiospores*** [100/2/2] (7.2) 8.1–9.9–11.5 (12.5) × (5.5) 6.9–8.0–9.3 (9.8) μm, *Q* = (1.01) 1.05–1.24–1.38 (1.47), *Qm* ± SD = 1.24 ± 0.086, nodulose, with 9–13 distinct rounded nodules, yellow to yellowish-brown in 5% KOH, usually containing a large oily droplet. Basidia 30.6–38.1 × 13.5–15.8 μm, subcylindrical to subclavate, mostly with four sterigmata 3–6 μm long, occasionally 2-sterigmate, thin-walled, colorless, with abundant contents, usually containing 1–4 oily droplets, occasionally transparent. ***Pleurocystidia*** 40–52–61 × 18–20–24 μm (*n* = 30), broadly fusiform to elongate-fusiform, occasionally utriform or guttulate, apex mostly obtuse, often encrusted with crystals, occasionally without crystals, with very few contents, base gradually tapering to truncate or obtuse, occasionally with a short pedicel, thick-walled, walls gradually thickening from base to apex, thickest at apex up to 3–4 μm, walls distinctly yellow, interior usually subhyaline, occasionally with very few contents. ***Cheilocystidia*** 39–44–50 × 15–19–22 μm (*n* = 30), similar to pleurocystidia, broadly fusiform, occasionally terete-clavate, walls up to 2–3 μm thick at apex, distinctly yellow. Lamellae edge with abundant paracystidia, 15–20 × 6–10 μm, subclavate to inflated-clavate, thin-walled, colorless, transparent, without contents. ***Caulocystidia*** 38–46 × 17–20 μm, present on the entire stipe surface (entirely pruinose), densely clustered on upper stipe, gradually decreasing downwards to base, narrowly elongate-fusiform or subclavate, apex obtuse, smooth, without crystals or occasionally with a few crystals, without contents; mostly with walls gradually thickening from base to apex, up to 2–3 μm thick at apex, occasionally thin-walled, walls distinctly yellow. Cauloparacystidia 17–25 × 8–14 μm, abundant, subglobose, clavate or subcylindrical, thin-walled, colorless, transparent, without contents. ***Hymenophoral trama*** 63–115 μm wide, subregular, composed of inflated hyphae 7–19 μm diam. ***Pileipellis*** a cutis, 80–140 μm thick, distinctly yellow, composed of elongate hyphae 4–13 μm wide and inflated cylindrical hyphae 15–24 μm wide; elongate hyphae thin-walled, pale yellow, transparent, smooth, occasionally rough; inflated hyphae thin-walled, pale yellow, rough. ***Pileal trama*** subregular, with elongate hyphae 5–14 μm wide in upper part, inflated hyphae 20–35 μm wide in lower part, smooth, thin-walled, colorless, transparent. ***Stipitipellis*** regularly arranged, hyphae cylindrical, 3–14 μm diam., smooth, occasionally rough, pale yellow. ***Stipe trama*** regularly arranged, hyphae elongate-cylindrical, 5–15 μm diam., thin-walled, smooth, colorless, transparent. ***Oleiferous hyphae*** common in pileus, stipe and hymenium, more abundant in pileus than in stipe and hymenium, 9–19 μm wide, with tuberculate nodules, pale yellow to yellow. ***Clamp connections*** present in all tissues.

##### Habitat.

In subtropical evergreen broad-leaved forests dominated by *Fagaceae*.

##### Geographical distribution.

Known only from Zhejiang Province, China.

##### Additional specimens examined.

China • Zhejiang Province: Hangzhou City, Lin’an District, Tianmu Mountain, 5 July 2018, in fagaceous forests, leg. T. Bau, FYG4020 (FCAS4451); Hangzhou City, Xihu District, Hangzhou Botanical Garden, in fagaceous forests, 20 July 2023, leg. Q.Q. Huang, FYG8421 (FCAS4453).

##### Remarks.

*Inocybe xihuensis* occurs from mid-June to early July in subtropical fagaceous forests in eastern China. It is characterized by a yellowish-brown pileus with a dark brown center, a stipe surface covered with granular pruina, basidiospores with 9–13 small rounded nodules, and hymenial cystidia that are obtusely fusiform to fusiform. Multigene phylogenetic analyses show that this species forms a sister group with a clade comprising *I. tiliae*, *I. sepiana*, *I. tenuiorparietalis*, and the *I. pallida*/*I. nobilis* complex. Among them, *I. sepiana* has smaller basidiomata that are brown to dark brown, angular-nodulose basidiospores with only weakly developed nodules, and occurs under *Pinus* (Bandini et al. 2022). *Inocybe tiliae* has basidiospores similar to those of *I. xihuensis* but differs in its more robust basidiomata, pileus with ochraceous-red tinges, fusiform to subutriform hymenial cystidia, and growth under *Tilia* (Franchi et al. 2016). *Inocybe tenuiorparietalis* has a dark ochraceous to ochre-brown pileus, angular basidiospores with weak nodules, and occurs at the edge of *Pinus densiflora* forests (Esteve-Raventós et al. 2022).

#### Inocybe tiliae

Taxon classificationFungiAgaricalesInocybaceae

Franchi, M. Marchetti & Papetti, Riv. Micol. 59(2): 102 (2016)

31B86779-24B3-51F7-8169-796BA95EEB05

##### Habitat.

Under *Tilia* or *Quercus* trees in Europe; in *Populus* or *Abies* forests in urban areas of northeastern China.

##### Geographical distribution.

Europe (type), China (Jilin).

##### Specimens examined.

China • Jilin Province: Changchun City, Helong Town, in *Populus* forests, 3 August 2023, leg. Y.G. Fan & W.J. Yu, FYG8482 (FCAS4450), FYG8490 (FCAS4446), FYG8492 (FCAS4445); • Songyuan City, Changling County, in *Populus* forests, 30 August 2023, leg. Y.G. Fan, FYG10253 (FCAS4447), FYG10262 (FCAS4443), FYG10266 (FCAS4449), FYG10270 (FCAS4448).

##### Remarks.

*Inocybe tiliae* is characterized by robust basidiomata and a reddish-tinged pileus in Europe (Franchi et al. 2016), where it was first discovered. However, the Chinese collections exhibit a brownish to yellowish-brown pileus and more slender stipes and occur mostly in artificial *Populus* plantations, as well as in *Abies* forests. Morphological variation was also observed among the Chinese specimens: e.g., FYG8482 has a flesh-pink-tinged stipe, whereas in FYG8490 and FYG8492, the stipes are less pinkish and the pileus color is mostly yellowish to earthy yellow. Multigene phylogenetic analyses show some genetic divergence between European and Chinese material, but with limited support. Therefore, the Chinese populations are currently treated as conspecific with *I. tiliae*. This is the first formal report of this species from China.

#### Inocybe dunensis

Taxon classificationFungiAgaricalesInocybaceae

P.D. Orton, Trans. Br. mycol. Soc. 43(2): 277 (1960)

0186C326-6F5C-5432-9985-A6714069658D

##### Habitat.

Growing on calcareous sandy soils, associated with *Salix repens* and *Pinus sylvestris* in Europe (Bandini et al. 2022). In China, it was found in *Populus* forests, producing basidiomata abundantly around mid-August.

##### Geographical distribution.

Europe (type); China (Jilin, Xizang Autonomous Region).

##### Specimens examined.

China • Jilin Province: Changchun City, Helong Town, in *Populus* forests, 17 August 2023, leg. W.J. Yu, FYG11420 (FCAS4461). Xizang Autonomous Region: Lhasa City, in *Populus* forests, 17 August 2023, leg. an amateur collector, FYG11505 (FCAS4460).

##### Remarks.

*Inocybe dunensis* is characterized by medium-sized, rather stout basidiomata, a yellowish-brown pileus, oblong spores with weak nodules, and hymenial cystidia that are broadly fusiform to sublageniform, with walls up to 5–6 µm thick (Bandini et al. 2022). The species is psammophilous and usually occurs on calcareous sandy soils. In this study, the species is reported for the first time from China, where it grows in planted *Populus* forests and produces basidiomata abundantly around mid-August. The Chinese material from Jilin has robust basidiomata, a yellowish-brown to straw-yellow pileus covered with appressed fibrillose scales, a yellowish-white stipe sometimes with flesh-colored tints, and a distinctly marginate bulb at the stipe base. Multigene phylogenetic analysis shows that this species is sister to *I. javorkae*. *Inocybe javorkae* is also a psammophilous species with robust basidiomata originally described from Hungary (Babos and Stangl 1985), but its pileus is brown to reddish-brown, the stipe base is black, and the spores are distinctly larger (11.7–16.0 × 6.1–8.9 µm, *Q* = 1.5–2.0; Bandini et al. 2022). It grows in inland calcareous sandy habitats, associated with *Festuca vaginata*, *Populus alba*, *Juniperus communis*, and *Pinus nigra* (Babos and Stangl 1985; Bandini et al. 2022).

***Inocybe pallida / Inocybe nobilis* complex**

**Habitat**. Occurs in forest margins and grasslands (presumably) or in coniferous litter under subalpine spruce forests in Europe, whereas in northwestern China it is found in subalpine shrublands.

**Geographical distribution**. Europe, China (Qinghai).

**Specimens examined**. China • Qinghai Province: Zhangye City, Ebao Town, in subalpine shrubs, 15 August 2024, leg. Y.G. Fan & J.R. Wang, FYG11067 (FCAS4442).

**Remarks**. *Inocybe pallida* was originally described by Velenovský (1920) from the Czech Republic, with angular-nodulose spores and a habitat in coniferous forests. Its holotype, however, is preserved in formaldehyde, and no DNA sequence could be obtained from it (Esteve-Raventós et al. 2018, 2022). *Inocybe nobilis* was introduced by Heim (1931) as *I. fibrosa* var. nobilis from montane coniferous forests, characterized by a very pale pileus and a clavate to subbulbous stipe (Heim 1931). Later, Alessio (1980) interpreted it as a species with brownish-pink basidiomata and a marginate bulb, based on material from lowland Mediterranean pine forests. The ITS sequence currently associated with *I. nobilis* (KX592683) originated from a coastal collection in Italy (Franchi et al. 2016) and shows 98.95% similarity to the sequence labeled *I. pallida sensu* Bizio & Marchetti (JF908198) (Esteve-Raventós et al. 2022). Morphologically, both taxa share robust basidiomata, a reddish-brown to ochraceous pileus, a marginate bulb, and clavate to fusiform hymenial cystidia (Franchi et al. 2016; Esteve-Raventós et al. 2022); no stable diagnostic characters separate them. Because molecular data cannot be obtained from the types of either name and their current species concepts rely heavily on subjective interpretations by later authors, this group is provisionally treated as a species complex.

In this study, only one collection from subalpine scrubland in Qinghai, China, was obtained. It has a robust stipe, a dark brown to chocolate-brown pileus, and a slightly pinkish stipe. Multigene phylogenetic analysis places this sample in the *I. pallida*/*I. nobilis* complex. Additional collections and more morphological and molecular data are needed to clarify the species boundaries within this complex.

## Discussion

In this study, an updated phylogeny of the Tiliae group was inferred. The analyses included taxa of the Tiliae clade and allied taxa from previous studies (Franchi et al. 2016; Esteve-Raventós et al. 2022; Bandini et al. 2022), together with newly collected specimens from subtropical and temperate China. The results led to the proposal of four new species (*I. qiongzhuensis*, *I. ravida*, *I. xihuensis*, and *I. zijinshanensis*) and three newly recorded taxa (*I. tiliae*, *I. dunensis*, and a lineage provisionally referred to as the *I. pallida*/*I. nobilis* complex). These discoveries substantially expand the known diversity of the group, which was previously known mainly from Europe, with only environmental or unpublished sequences hinting at its possible occurrence in Asia and North America.

Ecologically, the Chinese collections were found in diverse habitats ranging from subtropical broad-leaved forests to temperate *Populus* plantations and subalpine shrublands, indicating a broader ecological amplitude than the typical European habitats. The four new species were collected from subtropical evergreen broad-leaved forests in Central China, the world’s largest evergreen broad-leaved forest biome (Peng et al. 2026). This forest type is uniquely shaped by the Asian monsoon climate, with a tropical Asian floristic affinity at the generic level but dominated by endemic East Asian species at the species level (Deng et al. 2021, 2022; Hu et al. 2023; Zhou et al. 2023; Chen et al. 2024; Gao et al. 2025). Its formation is closely linked to the intensification of the Asian monsoon following the mid-Miocene uplift of the Himalaya–Tibet (Zhu and Tan 2024). Recent studies have identified Central China as a hidden global biodiversity hotspot for vascular plants (Feng et al. 2026), yet its fungal diversity remains largely unexplored (Yu et al. 2020; Zhao et al. 2022; Zhu et al. 2025). The discovery of new *Inocybe* species from this region underscores that Central China is not only a center of plant endemism but also a reservoir of previously undocumented fungal diversity.

Beyond expanding the known diversity and distribution of the group, the inclusion of these Chinese collections also improved the phylogenetic resolution of the Tiliae group. The former core clade (*I. tiliae*, *I. tenuiorparietalis*, the *I. pallida*/*I. nobilis* complex, *I. robertii*, and *I. sepiana*) remains monophyletic, but *I. robertii* is recovered within it with only limited support (PP/BS = –/72). Instead, a larger clade containing these five taxa, the Dunensis clade (*I. dunensis* and *I. javorkae*), and the four new Chinese species is fully supported (PP/BS = 1.00/100). This major clade, together with the Pseudohiulca clade and the Velata clade, forms the basis of the expanded Tiliae group defined below. The Velata clade is sister to the remaining lineages of the broad Tiliae group, whereas the Pseudohiulca clade is sister to the major clade. *Inocybe favrei*, previously compared with *I. sepiana* on morphological grounds, falls outside the broad Tiliae group and nests within the Praetervisae clade, consistent with Larsson et al. (2018).

In Bon’s (1998) traditional classification, the Tiliae clade was placed in sect. *Marginatae*, subsect. *Oblectabiles*. This subsection is characterized by nodulose to subangular spores, caulocystidia present at least on the upper two-thirds of the stipe, a predominantly bulbous base, and an often pinkish hue (Bon 1998). Within this framework, the Tiliae clade was further defined by robust basidiomata, a pileus ranging from pale ochraceous to chestnut brown, a pinkish stipe, and large heterodiametric spores with an average length exceeding 10 µm (Esteve-Raventós et al. 2022). However, when more closely related lineages are included, two features become problematic: spore size and the distribution of stipe pruinescence.

In the expanded group, the degree of stipe pruinescence varies markedly. The major clade and the Pseudohiulca clade have densely pruinose stipes, whereas the Velata clade shows a clear reduction: *I. velata* is almost devoid of pruina, and *I. decipiens sensu* Bandini is pruinose only in the upper part (Marchetti and Franchi 2008; Bandini et al. 2022). This character was traditionally used to separate sect. *Marginatae*, with entirely pruinose stipes, from sect. *Cortinatae*, with pruina only at the apex (Kühner 1933). However, molecular phylogenies have repeatedly shown that caulocystidia distribution is homoplastic and does not delimit monophyletic groups in *Inocybe* (Matheny 2005; Ryberg et al. 2010; Larsson et al. 2018; Bandini et al. 2022; Chen et al. 2024; Gao et al. 2025). The reduced pruinescence in the Velata clade is compatible with its basal position within the broad Tiliae group. This morphological divergence does not refute its phylogenetic placement, which is strongly supported by molecular evidence.

Spore size is also more variable than previously thought. Although most members of the expanded group have average spore lengths exceeding 10 µm, three of the new Chinese species (*I. qiongzhuensis*, *I. ravida*, and *I. xihuensis*), together with *I. pseudohiulca*, produce spores that fall partly below this threshold (Kühner 1933; Esteve-Raventós et al. 2022). Spore size thus remains diagnostically useful at the species level, but it is no longer a consistent feature of the clade as a whole.

Based on the phylogenetic evidence presented above, the Tiliae group is therefore redefined in a broad sense to include the major clade (ten species: the former core lineage, *I. robertii*, the Dunensis clade, and the four new Chinese species), the Pseudohiulca clade, and the Velata clade (*I. velata* and *I. decipiens sensu* Bandini). Under this expanded circumscription, the broad Tiliae group comprises 12 species and two species complexes in total. The broad Tiliae group is defined by a combination of features: robust basidiomata, a yellowish-ochraceous to dark brown pileus with appressed fibrils, a stipe often with pinkish tones, a bulbous or marginate stipe base, a stipe that is either entirely pruinose or at least pruinose in the upper half, and often a strong spermatic odor. Although variation occurs, the combination of these features, together with strong phylogenetic support, consistently distinguishes the broad Tiliae group from other lineages within sect. *Marginatae* and provides a more reliable framework for understanding the diversity and evolution of the group.

Four new species of *Inocybe* are described from different parts of China in the present paper, suggesting that the diversity of *Inocybe* in China remains insufficiently known, as is also the case for many other groups of macrofungi in China (Dai et al. 2021; Li et al. 2024; Wang et al. 2025; Song et al. 2025; Cui et al. 2026; Zhao et al. 2026). Therefore, more new taxa of *Inocybe* are likely to be discovered through further investigations.

## Supplementary Material

XML Treatment for Inocybe qiongzhuensis

XML Treatment for Inocybe ravida

XML Treatment for Inocybe zijinshanensis

XML Treatment for Inocybe xihuensis

XML Treatment for Inocybe tiliae

XML Treatment for Inocybe dunensis

## Additional information

### Artificial Intelligence (AI) use

The authors accept full responsibility for the content of the manuscript, including the disclosure of any use of AI.

Regarding the use of AI in the preparation of this manuscript, the authors declare the following: DeepSeek

Used for: Language, style and writing

Artificial intelligence tools (Deepseek, https://www.deepseek.com) were only used for basic grammatical checking.

### Author ORCIDs

Y.-G. Fan https://orcid.org/0000-0002-3028-9752

Y.-R. Xu https://orcid.org/0009-0004-8382-1636

Y. Li https://orcid.org/0009-0004-8068-8470

C. Chang https://orcid.org/0000-0001-5386-6754

L.-S. Deng https://orcid.org/0000-0002-8210-3884

W.-J. Yu https://orcid.org/0000-0002-0147-3091

### Data availability

All of the data that support the findings of this study are available in the main text or Supplementary Information.

## Supplementary materials

Supplementary material 1 Taxon, sequences, and their information used in the phylogenetic analyses Data type doc https://binary.pensoft.net/file/1754401This dataset is made available under the Open Database License (http://opendatacommons.org/licenses/odbl/1.0/). The Open Database License (ODbL) is a license agreement intended to allow users to freely share, modify, and use this Dataset while maintaining this same freedom for others, provided that the original source and author(s) are credited. Yu-Guang Fan, Yan-Ru Xu, Ying Li, Cheng Chang, Lun-Sha Deng, Wen-Jie Yu
