# Supplementary material for: Molecular phylogeny of the Tiliae group (Inocybe sect. Marginatae) and delimitation of the group with new discoveries
Source: MycoKeys. 2026 Aug 25;139:109–38. doi: 10.3897/mycokeys.139.204873 (PMC13535506; doi:10.3897/mycokeys.139.204873)
Supplement: Supplementary file 1 [file mycokeys-139-109-s001.doc]

**Table S1. Taxon, sequences, and their information used in the phylogenetic analyses.**

| Taxon | Collection no. and/or specimen no. | Locality | GenBank acc. no. ITS | Genbank acc. no. LSU | Genbank acc. no. *rpb2* | References |
| --- | --- | --- | --- | --- | --- | --- |
| *I. albodiscoides* | JK189 | Washington | ON116980 | ON113316 | ON221348 | Direct Submission |
| *I. asterospora* | EL100-14 | Sweden | MN296110 | MN296110 | ― | Direct Submission |
| *I. avellaneorosea* | AH 23468 | Spain | ON994217 | ON994217 | ― | Esteve-Raventós et al. (2022) |
| *I. avellaneorosea* | A5058255 | Spain | ON994218 | ON994218 | ― | Esteve-Raventós et al. (2022) |
| *I. avellaneorosea* | AH 51879 (holotype) | France | ON994216 | ON994216 | ― | Esteve-Raventós et al. (2022) |
| *I. candidipes* | BK-24-July-99-7 (paratype) | USA | AY239019 | AY239019 | AY239029 | Direct Submission |
| *I. decipiens* | DB16-6-12-E60- Eimann | Germany | OP164018 | OP164018 | ― | Bandini et al. (2022) |
| *I. decipiens* | DB3-9-10-Dondl | Germany | OP164017 | ― | ― | Bandini et al. (2022) |
| *I. decipiens* | iNAT196289125 | USA-Florida | PX139608 | ― | ― | Direct Submission |
| *I. dunensis* | KR-M-0038114 | Netherlands | OP164019 | OP164019 | ― | Bandini et al. (2022) |
| *I. dunensis* | DB15-8-14-23 | Germany | OP164020 | ― | ― | Bandini et al. (2022) |
| *I. dunensis* | FYG11420 (FCAS4461) | China | PZ555311 | PZ56592 | PZ780768 | This study |
| *I. dunensis* | FYG11505 (FCAS4460) | China | PZ555310 | PZ565924 | PZ780767 | This study |
| *I. dunensis* | 237 | Italy | JF908092 | ― | ― | Osmundson et al. (2013) |
| *I. favrei* | G J. Favre 122a (lectotype of. *I. taxocystis*) | Switzerland | JN580884 | ― | ― | Kokkonen and Vauras (2012) |
| *I. favrei* | Bon84095 (type) | France | KY033786 | ― | ― | Larsson et al. (2018) |
| *I. favrei* | EL5706 | Sweden | FN550886 | FN550886 |  | Ryberg et al. (2010) |
| *I. flavipes* | MR00383 (holotype) | Togo | NR184904 | MN097889 | MW080915 | Aïgnon et al. (2022) |
| *I. flavipes* | HLA0363 | Benin | MT994601 | ― | ― | Direct Submission |
| *I. javorkae* | SMNS-STU- F-0901704 | Germany | OP164078 | OP164078 | ― | Direct Submission |
| *I. javorkae* | BP 77256 A257 (holotype) | Hungary | ON359948 | ― | ― | Direct Submission |
| *I. javorkae* as  *I. dunensis* | 243 | Italy | JF908093 | ― | ― | Osmundson et al. (2013) |
| *I. javorkae* as  *I. dunensis* | 21679 | Italy | JF908262 | ― | ― | Osmundson et al. (2013) |
| *I. kohistanensis* | LAH 35001 | Pakistan | NR_153155 | ― | ― | Jabeen et al. (2016) |
| *I. lineata* | DED8048 | Thailand | ― | GQ892958 | KM245999 | Horak et al. (2015) |
| *I. lineata* | DED8019 (holotype) | Thailand | EU56986 | EU56986 | ― | Matheny et al. (2009) |
| *I. lucida* | AH_50955 | Spain | ON994205 | ― | ― | Direct Submission |
| *I. lucida* | AH_25011 | Spain | ON994210 | ON994210 | ― | Esteve-Raventós et al. (2022) |
| *I. nobilis* | Rebaudengo-27-10-1982 | Italy | KX592683 | ― | ― | Franchi et al. (2016) |
| *I. nobilis* | DB8-10-11-6 | Germany | OP164028 | OP164028 | ― | Bandini et al. (2022) |
| *I. oblectabilis* | JV32005 | Italy | ON754370 | ON754370 | ― | Esteve-Raventós et al. (2022) |
| *I. oblectabilis* | M:0222614 (epitype) | Italy | KM873365 | ― | ― | Marchetti et al. (2014) |
| *I. pallida* | 21493 | Italy | JF908198 | ― | ― | Osmundson et al. (2013) |
| *I. pallida-nobilis* | FYG11067 (FCAS4442) | China | PZ555296 | PZ565907 | PZ780751 | This study |
| *I. panamica* | AC59 | Panama | ON116987 | ON113322 | ON221353 | Matheny et al. (2022) |
| *I. phaeosticta* | PAM05091310 | France | HQ586859 | HQ641102 | MH577435 | Matheny unpublished |
| *I. phaeosticta* | PAM05072305 | France | HQ586873 | HQ641110 | MH577436 | Matheny unpublished |
| *I. piceae* | M 0138785 (holotype) | Italy | KX592682 | ― | ― | Direct Submission |
| *I. piceae* | EL215-08 | Sweden | ON754366 | ON754366 | ― | Esteve-Raventós et al. (2022) |
| *I. piceae* var. *tenuiorparietalis* | EL408-17 | Sweden | ON754369 | ― | ― | Direct Submission |
| *I. pseudohiulca* | G053288 | France | ON845750 | ― | ― | Esteve-Raventós et al. (2022) |
| *I. qiongzhuensis* | FYG10497 (FCAS4459, holotype) | China | PZ555309 | PZ565923 | PZ780766 | This study |
| *I. qiongzhuensis* | FYG10499 (FCAS4457) | China | PZ555307 | PZ565921 | PZ780764 | This study |
| *I. qiongzhuensis* | FYG10683 (FCAS4458) | China | PZ555308 | PZ565922 | PZ780765 | This study |
| *I. ravida* | FYG10518 (FCAS4462, holotype) | China | PZ555312 | PZ565926 | PZ780769 | This study |
| *I. ravida* | FYG10668 (FCAS4463) | China | PZ555313 | PZ565927 | PZ780770 | This study |
| *I. ravida* | FYG12694 (FCAS4465) | China | PZ555315 | ― | ― | This study |
| *I. ravida* | FYG12700 (FCAS4466) | China | PZ555316 | ― | ― | This study |
| *I. rivularis* | JV-3610F (paratype) | Finland | JN580885 | ― | ― | Direct Submission |
| *I. robertii* | Guten990923 (epitype) | Sweden | AM882979 | ― | ― | Ryberg et al. (2008) |
| *I. robertii* | AH45182 | Spain | ON754365 | ON754365 | ― | Direct Submission |
| *I. robertii* (as *I.* cf. *oblectabilis*) | KRMO044689 | Germany | MTO05874 | ― | ― | Poyet et al. (2019) |
| *I. robertii* (as *I.* cf. *oblectabilis*) | KRM0044734 | Germany | MT005876 | ― | ― | Direct Submission |
| *I. sepiana* | SMNS-STU- F-0901688 | Germany | OP164103 | OP164103 | ― | Bandini et al. (2022) |
| *I. sepiana* | SMNS-STU- F-0901705 | Germany | OP164104 | OP164104 | ― | Bandini et al. (2022) |
| *I. sepiana* | SMNS-STU- F-0901687 (holotype) | Germany | OP164056 | OP164056 | ― | Bandini et al. (2022) |
| *I. sepiana* | SMNS-STU- F-0901718 | Austria | OP164100 | OP164100 | ― | Bandini et al. (2022) |
| *I.* sp. | FYG11061 (FCAS4464) | China | PZ555314 | PZ565928 | PZ780771 | This study |
| *I. tabacina* | HMUT-M1287 | China | ― | ON430593 | ON470149 | Direct Submission |
| *I. tabacina* | PAM05071302 | France | HQ586865 | HQ641106 | MH577481 | Matheny unpublished |
| *I. tenuiorparietalis* | M0216705 (holotype) | Sweden | ON754368 | ― | ― | Esteve-Raventós et al. (2022) |
| *I. tenuiorparietalis* | Blom9909 | USA- Florida | PQ892450 | PQ892450 | ― | Direct Submission |
| [*I. tenuiorparietalis* as](../Inocybesp.as)  *I. oblectabilis* | BJ920908 | Sweden | AM882831 | AM882831 | ― | Ryberg et al. (2008) |
| *I. tenuiorparietalis* as *I. oblectabilis* | MB980915 | Sweden | AM882833 | ― | ― | Ryberg et al. (2008) |
| *I. tiliae* | KR-M-0038132 | Germany | OP164036 | OP164036 | ― | Bandini et al. (2022) |
| *I. tiliae* | SMNS-STU- F-0900994 | Germany | OP164070 | OP164070 | ― | Bandini et al. (2022) |
| *I. tiliae* | FYG10253 (FCAS4447) | China | PZ555298 | PZ565911 | PZ787497 | This study |
| *I. tiliae* | FYG10262 (FCAS4443) | China | PZ555297 | PZ565908 | PZ780752 | This study |
| *I. tiliae* | FYG10266 (FCAS4449) | China | PZ555300 | PZ565912 | PZ780756 | This study |
| *I. tiliae* | FYG10270 (FCAS4448) | China | PZ555299 | PZ565912 | PZ780755 | This study |
| *I. tiliae* | FYG8482 (FCAS4450) | China | ― | PZ565914 | PZ780757 | This study |
| *I. tiliae* | FYG8490 (FCAS4446) | China | ― | PZ565910 | PZ780754 | This study |
| *I. tiliae* | FYG8492 (FCAS4445) | China | ― | PZ565909 | PZ780753 | This study |
| *I. tiliae* | AMB 18103 (holotype) | Italy | KX592681 | ― | ― | Franchi et al. (2016) |
| *I. tundrae* | M0216707 (holotype) | Sweden | MK153627 | ― | ― | Cripps et al. (2020) |
| *I. vagata* | PBM3187 | Australia | KP641639 | KP171091 | KM656135 | Matheny and Bougher (2017) |
| *I. velata* | MCVE 23506 (holotype) | Italy | OP164047 | ― | ― | Bandini et al. (2022) |
| *I. velata* | SMNS-STU- F-0901721 | Germany | OP164085 | OP164085 | ― | Bandini et al. (2022) |
| *I. xihuensis* | FYG4020 (FCAS4451) | China | PZ555301 | PZ565915 | PZ780758 | This study |
| *I. xihuensis* | FYG8385 (FCAS4452, holotype) | China | PZ555302 | PZ565916 | PZ780759 | This study |
| *I. xihuensis* | FYG8421 (FCAS4453, holotype) | China | PZ555303 | PZ565917 | PZ780760 | This study |
| *I. zijinshanensis* | FYG8388 (FCAS4455) | China | PZ555304 | PZ565919 | PZ780762 | This study |
| *I. zijinshanensis* | FYG8412 (FCAS4454, holotype) | China | PZ555304 | PZ565918 | PZ780761 | This study |
| *I. zijinshanensis* | FYG9816 ( FCAS4456) | China | PZ555306 | PZ565920 | PZ780763 | This study |
| *Nothocybe distincta* | CAL 1310 | India | NR173156 | NG057278 | KX171345 | Latha et al. (2016) |
| *N. distincta* | ZT 9250 | India | ― | EU604546 | EU600904 | Latha et al. (2016) |
